# Proteomic identification of the lactate dehydrogenase A in a radioresistant prostate cancer xenograft mouse model for improving radiotherapy

**DOI:** 10.18632/oncotarget.12368

**Published:** 2016-09-30

**Authors:** Jingli Hao, Peter Graham, Lei Chang, Jie Ni, Valerie Wasinger, Julia Beretov, Junli Deng, Wei Duan, Joseph Bucci, David Malouf, David Gillatt, Yong Li

**Affiliations:** ^1^ Cancer Care Centre, St George Hospital, Kogarah, NSW 2217, Australia; ^2^ St George and Sutherland Clinical School, Faculty of Medicine, University of New South Wales, Sydney, NSW 2052, Australia; ^3^ Department of Obstetrics and Gynecology, The First Affiliated Hospital of Zhengzhou University, Zhengzhou, Henan 450052, China; ^4^ Bioanalytical Mass Spectrometry Facility, Mark Wainwright Analytical Centre, Sydney, NSW 2052, Australia; ^5^ School of Medical Sciences, Sydney, NSW 2052, Australia; ^6^ SEALS, Anatomical Pathology, St George Hospital, Kogarah, NSW 2217, Australia; ^7^ School of Medicine, Deakin University, Waurn Ponds, Victoria 3217, Australia; ^8^ Department of Urology, St George Hospital, Kogarah, NSW 2217, Australia; ^9^ Australian School of Advanced Medicine, Macquarie University, Sydney, NSW 2019, Australia

**Keywords:** prostate cancer, proteomics, glycolysis, LDHA, radiotherapy

## Abstract

Radioresistance is a major challenge for prostate cancer (CaP) metastasis and recurrence after radiotherapy. This study aimed to identify potential protein markers and signaling pathways associated with radioresistance using a PC-3 radioresistant (RR) subcutaneous xenograft mouse model and verify the radiosensitization effect from a selected potential candidate. PC-3RR and PC-3 xenograft tumors were established and differential protein expression profiles from two groups of xenografts were analyzed using liquid chromatography tandem-mass spectrometry. One selected glycolysis marker, lactate dehydrogenase A (LDHA) was validated, and further investigated for its role in CaP radioresistance. We found that 378 proteins and 51 pathways were significantly differentially expressed between PC-3RR and PC-3 xenograft tumors, and that the glycolysis pathway is closely linked with CaP radioresistance. In addition, we also demonstrated that knock down of LDHA with siRNA or inhibition of LDHA activity with a LDHA specific inhibitor (FX-11), could sensitize PC-3RR cells to radiotherapy with reduced epithelial-mesenchymal transition, hypoxia, DNA repair ability and autophagy, as well as increased DNA double strand breaks and apoptosis. In summary, we identified a list of potential RR protein markers and important signaling pathways from a PC-3RR xenograft mouse model, and demonstrate that targeting LDHA combined with radiotherapy could increase radiosensitivity in RR CaP cells, suggesting that LDHA is an ideal therapeutic target to develop combination therapy for overcoming CaP radioresistance.

## INTRODUCTION

Radiotherapy (RT) is a standard treatment option for both organ-confined and regionally advanced prostate cancer (CaP). Despite more and more effective advances in radiation delivery procedures, about 50% CaP patients undergoing RT suffer from relapse (recurrence) within 5 years of treatment [1]. Radioresistance (the failure to RT) is a major challenge for the current CaP RT. The mechanisms of cancer radioresistance are very complicated and affected by many factors, which severely affect radiation efficacy. One reason for these failures following RT is due to the intrinsic radioresistance of a subpopulation of clones within the tumor [2] while another reason could be the acquired radioresistance during RT [3, 4]. Current markers used in clinics are not sufficient to separate radiosensitive CaP from radioresistant (RR) CaP to predict its radiation response and develop a personalized treatment. Therefore, it is important to identify therapeutic targets associated with CaP radioresistance and develop novel adjuvant treatments to cure the disease. If the potential targets for radiosensitization are identified and further validated, it will achieve a more favorable therapeutic ratio in clinics.

Due to considerable genetic, behavioral, and environmental heterogeneity, it is very difficult to discover and verify cancer biomarkers directly in human samples. Mouse model can be conducted under stringent genetic and environmental control for biomarker discovery and verification [5, 6]. The mouse model derived biomarkers, after validation in human patient' sera, have been applied to develop multifactorial predictors of survival of castration-resistant CaP (CRPC) [7]. Therefore, the application of mouse models is highly promising for expanding our understanding of radioresistance, biomarker identification, and thereby moving closer to enhancing the prediction of radiosensitivity, and improving the treatment of RR cancer patients.

Using a low dose fractionated radiation treatment, we have recently developed CaP-RR cell lines with increased colony formation, invasion ability, sphere formation capability and enhanced epithelial-mesenchymal transition (EMT) and cancer stem cell (CSC) phenotypes and the activation of the PI3K/Akt/mTOR signaling pathway [8]. In the current study, one of the CaP-RR cell lines-PC-3RR, which was developed mimicking clinical conditions and representing the source of CaP recurrence after RT, was implanted subcutaneously in non-obese diabetic/severe combined immunodeficiency (NOD/SCID) mice and allowed to develop xenograft tumors *in vivo*. Comparing protein profiles of the RR tumor xenografts to the radiosensitive tumor xenografts provides an ideal model to investigate biomarkers and signaling pathways associated with CaP radioresistance.

In this study, we firstly identified differentially expressed proteins (DEPs) and a panel of pathways associated with CaP radioresistance from PC-3 and PC-3RR xenografts using liquid chromatography tandem-mass spectrometry (LC-MS/MS) technique and Ingenuity Pathway Analysis (IPA). In addition, a glycolysis marker lactate dehydrogenase A (LDHA), which is among the significant DEPs and sits in one of the most significantly deregulated pathways in radioresistance, was further validated in CaP-RR cell lines and PC-3RR xenograft tumors. LDHA is a main metabolic enzyme for lactate production which is a terminal product from glycolysis and plays an essential role in the glycolysis. We demonstrated that silencing of LDHA using the small interfering RNA (siRNA) or inhibition of LDHA with a small molecular inhibitor (FX-11) combined with RT reversed radioresistance in PC-3RR cells. Our findings have significant implications for developing novel therapies to overcome radioresistance and improve current CaP RT.

## RESULTS

### Establishment of the PC-3 and PC-3RR s.c xenograft models

**Figure 1:**
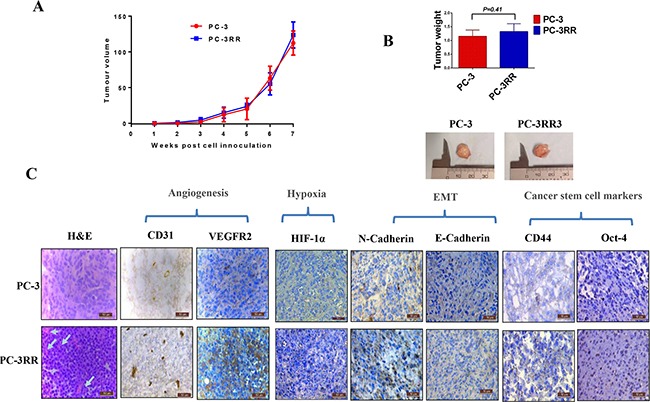
Growth of PC-3 and PC-3RR tumors *in vivo* and IHC for vasculature, hypoxia, EMT and CSC markers in animal xenografts **A.** The growth rates of PC-3 and PC-3RR s.c tumors. PC-3 and PC-3RR tumors were allowed to grow for 7 weeks. No significant difference was found for tumor growth between two models at all time points (*p*>0.05). **B.** At the end of the experiment, the tumor weights from PC-3 and PC-3RR groups of mice did not show significant difference (n=5, mean±SD, *p*>0.05). Representative images are shown for the tumor sizes in the two groups of mice. **C.** Histology and IHC evaluation of PC-3 and PC-3RR tumors. The cellular patterns and vasculatures (indicated by the white arrows) are shown in the H&E staining. CD31, VEGFR2, HIF-1α, N-Cadherin, CD44 and Oct-4 were upregulated in PC-3RR xenografts while E-Cadherin was downregulated in PC-3RR xenografts. Brown indicates positive staining while blue indicates nuclei. Magnification x 400 in all images. Scale bar=50μm

PC-3 and PC-3RR subcutaneous (s.c) xenograft tumors were established and allowed to grow up to 7 weeks. The growth rate of PC-3 and PC-3RR tumors at all time points and their tumor sizes at the end of the experiment did not show statistical difference (*p*>0.05) (Figure 1A and 1B). The tumor xenografts from these mice were collected at the end of experiment for immunohistochemistry (IHC) staining as well as for proteomics analysis.

### Histological and IHC differences observed between PC-3 and PC-3RR s.c xenograft tumors

The histology (H&E staining) results from two animal xenografts indicate that the density of blood vessels in the PC-3RR tumors (as shown by the arrows) was increased compared to the PC-3 tumors (Figure 1C), suggesting the existence of higher levels of angiogenesis in PC-3RR model. The increased vasculature (angiogenesis) was further confirmed by CD31 and VEGFR2 staining (Figure 1C). Hypoxia marker HIF-1α was also found to be increased in the PC-3RR tumors compared to the PC-3 tumors (Figure 1C). In addition, we also found enhanced EMT (increased N-Cadherin and reduced E-Cadherin) and CSC phenotypes (CD44 and Oct-4) in the PC-3RR xenograft tumors compared to the PC-3 control tumor (Figure 1C), which is consistent with our previous *in vitro* studies with CaP-RR cell lines [8]. The immunostaining intensity of IHC for CD31, VEGFR2, HIF-1α, EMT and CSC markers is summarized in Supplementary Table S1. These results suggest that PC-3RR xenograft tumor model retains phenotypic features of *in vitro* PC-3RR cells [8] and is suitable for proteomic analysis of CaP-RR biomarkers.

### Protein identification and quantification in PC-3 and PC-3RR xenograft tumors

**Figure 2:**
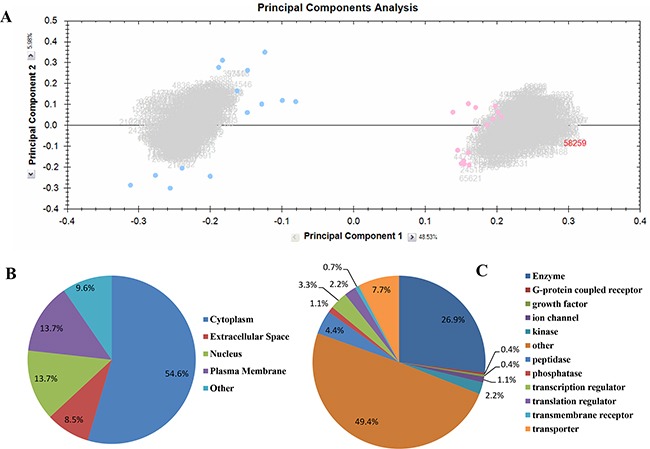
Proteomics analysis using Progenesis QI and IPA **A.** Principal component analysis (PCA) of global protein profiles in PC-3 and PC-3RR xenograft tumors. Principal component 1 (PC1) and principal component 2 (PC2) were plotted. The blue data points (Left) represent the samples from PC-3RR xenograft tumors, and the pink data points (Right) represent the samples from PC-3 xenograft tumors. The blue and red clusters were well separated. **B.** Ontology analysis of the significantly altered proteins between PC-3 and PC-3RR xenografts. Cellular compartments of the differentially expressed proteins in PC-3RR compared to PC-3 control xenografts. **C.** Ontology analysis of the significantly altered proteins between PC-3 and PC-3RR xenografts. Functions of the differentially expressed proteins in PC-3RR compared to PC-3 control xenografts.

To investigate the DEPs in PC-3 and PC-3RR xenograft tumors, multivariate analysis of protein expression was performed using principal components analysis (PCA), according to abundance variation. It was demonstrated that PC-3 tumors clustered (the pink spots- left side) while PC-3RR tumors clustered (the blue spots-right side) (Figure 2A). This demonstrates that 49% of the differences observed between these phenotypes can be attributed to the PC-3 xenografts vs PC-3RR xenografts. ANOVA *p*-values were used to determine DEPs. There were 378 proteins identified to be significantly differentially expressed between PC-3 and PC-3RR tumor xenograft samples (*p*<0.05) (Supplementary Table S2).

### Network analysis and top canonical pathway terms in PC-3 and PC-3RR xenograft tumors

IPA was used to reveal the signaling networks and metabolic pathways enriched in the DEPs. Data from all DEPs were submitted in Ingenuity software for pathway analysis. Fifty one pathway terms that are significantly deregulated in PC-3RR tumours were identified (Supplementary Table S3). Thirty-seven of those are associated with CaP (Supplementary Table S3). The important pathways related with CaP include Glycolysis Metabolism, VEGF, and Epithelial Adherens Junction signaling pathways. Using IPA, the DEPs were clustered based on locations and functions performed (Figure 2B, 2C). Of the 378 DEPs identified from PC-3 and PC-3RR paired xenografts, 271 proteins were mapped to pathways which were overly enriched in RR samples. The origin of these enriched pathway proteins includes 148 cytoplasm (54.6%) and 37 nucleus (13.7%), 37 plasma membrane (13.7%) and 23 extracellular space (8.5%) (Figure 2B). The main cellular molecular functions of these DEPs include 73 enzymes (26.9%), 21 kinase/peptidase/ phosphatases (7.7%), 21 transporters (7.7%), 9 transcription regulators (3.3%), and 6 translation regulators (2.2%) (Figure 2C). These results indicate the DEPs identified have multiple functional roles in CaP radioresistance.

### Examination of glycolysis pathway proteins in PC-3 and PC-3RR xenograft tumors

**Figure 3:**
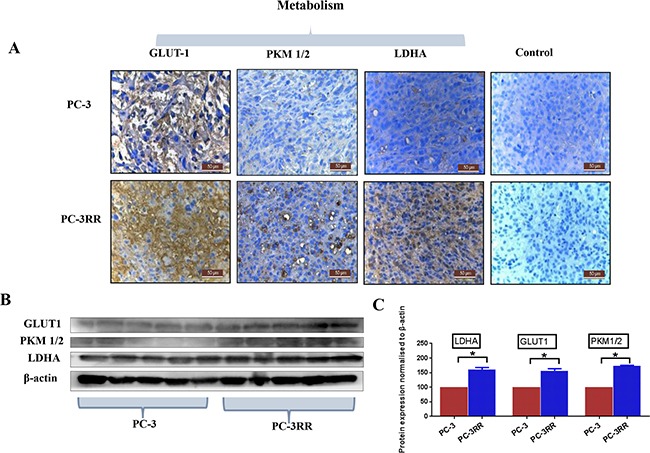
Validation of glycolysis proteins in PC-3 and PC-3RR xenografts **A.** Validation of glycolysis proteins using IHC. The glycolysis pathway proteins GLUT-1, PKM1/2 and LDHA, were upregulated in PC-3RR xenografts compared to PC-3 xenografts. Brown indicates positive staining while blue indicates nuclei. Magnification x 400 in all images. **B.** Validation of glycolysis protein using Western blot. The upregulation of glycolysis pathway proteins GLUT-1, PKM1/2 and LDHA in PC-3RR xenografts were confirmed by Western blot. β-actin was used as a loading control. **C.** Quantification of Western blot results from PC-3 and PC-3RR xenografts. The results were normalized by the level of β-actin. Results are expressed as mean ± SD (n=3). “*” indicates: *p*<0.05. Scale bar=50μm.

As glycolysis pathway was identified as an important signaling pathway from the proteomic analysis, we further validated the key proteins of this pathway in PC-3 and PC-3RR xenograft tumors by IHC and Western blot. We found that the expression of the key glycolysis markers (GLUT-1, PKM1/2 and LDHA) were significantly increased in PC-3RR xenografts compared with PC-3 xenografts and no positive staining for the detected markers was observed in the control sections (Figure 3A). The immunostaining intensity of glycolysis markers from IHC is summarized in Supplementary Table S1. The IHC results from PC-3 and PC-3RR xenograft tumors were confirmed by Western blot (Figure 3B, 3C). These results further support that the glycolysis pathway is associated with CaP radioresistance.

### Expression of LDHA in CaP RR cell lines and PC-3RR xenograft tumors

**Figure 4:**
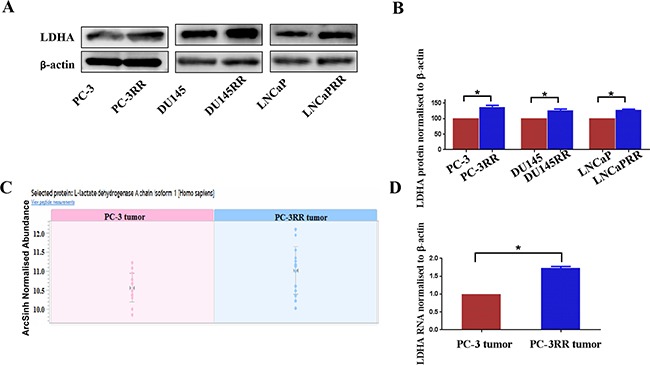
Expression of LDHA in CaP-RR cell lines and PC-3RR xenograft model **A.** LDHA protein expression in CaP-RR cell lines. Higher levels of LDHA expression were seen in CaP-RR cell lines (PC-3RR, DU145-RR and LNCaP-RR) compared to their matching control cells.β-actin was used as a loading control. **B.** Quantification of Western blot results from CaP and CaP-RR cell lines. The results were normalized by the level of β-actin. Results are expressed as mean ± SD (n=3) (*p*<0.05). **C.** Abundance of LDHA was shown using Progenesis IQ software. **D.** Validation of LDHA mRNA in PC-3 and PC-3RR xenografts. LDHA mRNA levels were detected using qRT-PCR and was normalized to β-actin level. Significantly increased LDHA mRNA was associated with PC-3RR xenografts (*p*<0.05). “*” indicates: *p*<0.05.

To further study the importance of glycolysis in CaP radioresistance, LDHA was chosen as a candidate for the functional validation. We demonstrated that increased expression of LDHA protein was found in CaP-RR (PC-3RR, DU145RR and LNCaPRR) cells compared with CaP control cells (Figure 4A). The quantification of LDHA Western blot results from CaP and CaP-RR cell lines was presented in Figure 4B. The results were normalized by the level of β-actin and expressed as mean ± SD (n=3) (*p*<0.05). In addition, we also demonstrated the up-regulation of the LDHA protein in PC-3RR tumors by re-examining the protein levels of LDHA from LC-MS/MS results using Progenesis QI software, (Figure 4C). Furthermore, increased expression of LDHA mRNA was also confirmed in PC-3RR xenograft tumors compared with PC-3 tumors using qRT-PCR (Figure 4D). All these results indicate LDHA could be a potential therapeutic target for CaP RT.

### Radiosensitisation effect after knock down (KD) or inhibition of LDHA in PC-3RR cells

**Figure 5:**
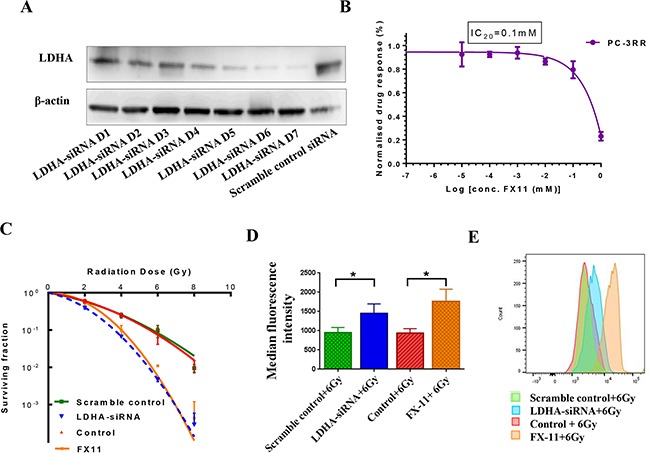
siRNA knocking down LDHA, MTT assay for FX-11 inhibitor, colony formation assay and ROS assay for single RT and combination treatments **A.** siRNA knocking down LDHA. LDHA protein expression was observed at 1-7 days post LDHA-siRNA and scr-siRNA transfection. **B.** MTT assay for FX-11 inhibitor. PC-3RR cells were treated with FX-11 in final concentrations of 0-1 mM. The IC_20_ value was calculated to be 0.1mM. **C.** Colony formation assay. PC-3RR cells were treated with combination of LDHA-siRNA (scr-siRNA) or FX-11 (vehicle) with RT (2, 4, 6, and 8 Gy) for analysis of colony-forming efficiency. Survival fractions were significantly reduced in PC-3RR cells after LDHA-siRNA+RT or FX-11+RT treatments compared to control combination treatments (*p*<0.05 at 4, 6, 8 Gy). **D.** ROS analysis by flow cytometry. After specific combination treatments or control combination treatments, intracellular ROS production was measured by flow cytometry. As shown by the median fluorescent intensity, significantly higher levels of ROS were detected in combination of LDHA-siRNA and RT or FX-11 and RT in PC-3RR cells compared with scr-siRNA+RT or vehicle+RT (*p*<0.05). **E.** The representative image of histograms of ROS after single RT or combination treatments. All results were from three independent experiments (n=3). “*” indicates: *p*<0.05

To investigate the role of LDHA in CaP RT, LDHA was knocked down in PC-3RR cells using siRNA (LDHA-siRNA). The level of LDHA expression after KD was examined by Western blot. It was found that after 4 days of siRNA transfection, the cells became irregular and tended to detach from the bottom of the flask (data not shown) and that the maximum level of KD for LDHA was obtained at day 7 (D7) post siRNA transfection (Figure 5A). Although the maximum KD effect was achieved at day 7, all experiments were performed at day 4 post siRNA transfection to retain the viability of cells. In addition to the LDHA-siRNA, the LDHA specific inhibitor FX-11 was also used to inhibit LDHA activity *in vitro*. The dose response of PC-3RR cells to FX-11 treatment was determined using an MTT assay (Figure 5B). IC_20_ (0.1mM) for FX-11 from MTT assay was used for all following experiments.

To study the radiosensitization effect of KD or inhibition of LDHA using siRNA or FX-11 treatment, single RT or the combination of LDHA-siRNA (inhibitor FX-11) and RT treatments were performed for clonogenic ability (Figure 5C). It was found that the survival fractions at 4, 6, 8 Gy were significantly different between combinations of LDHA-siRNA/FX-11 inhibitor with RT and single RT controls (scr-siRNA or treatment with the equal volume of DMSO) in PC-3RR cells, respectively (*p*<0.05) (n=3), suggesting that KD or inhibition of LDHA led to markedly reduced clonogenic ability and sensitized PC-3RR cells to radiation.

### Effects of LDHA KD or inhibition on reactive oxygen species (ROS)

As ROS plays an important role in radiation-induced cellular damage and cancer radioresistance [9], we investigated whether ROS is involved in the LDHA-siRNA and FX-11 mediated radiosensitization effects. Our results indicated that LDHA-siRNA or FX-11 treatment, combined with 6 Gy radiation, caused a significant increase of median fluorescence intensity (Figure 5D) and ROS levels (Figure 5E), compared to control groups in PC-3RR cells, respectively (*p*<0.05).

### Effects of LDHA KD or LDHA inhibition on EMT, hypoxia, DSB, DNA repair pathway, apoptosis and autophagy proteins

To further investigate mechanisms of combination of LDHA-siRNA or FX-11 and RT on CaP radiosensitization, the representative markers associated with EMT, hypoxia, DSB (DNA double-strand breaks), NHEJ (non-homologous end-joining inhibitor), apoptosis and autophagy were assessed in PC-3RR cells with different treatments and control treatments including: scr-siRNA control, scr-siRNA+6 Gy RT, LDHA-siRNA, LDHA-siRNA+6 Gy RT, vehicle control, vehicle control+6Gy RT, FX-11 and FX-11+6 Gy RT using Western blot.

**Figure 6:**
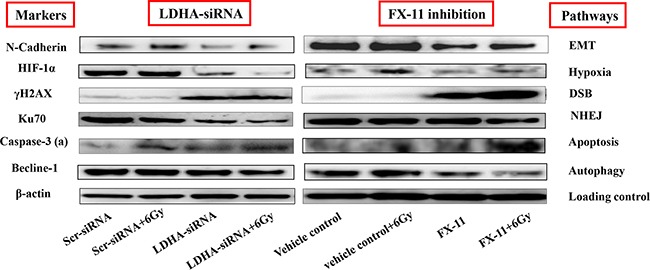
The putative mechanisms of radiosensitisation caused by LDHA-siRNA downregulation or FX-11 inhibition in PC-3RR cells Expression of N-Cadherin (EMT), HIF-1α (hypoxia), γH2AX (DNA DSB), Ku70 (DNA repair), Caspase-3 (active) (apoptosis) and Becline-1 (autophagy) was studied by Western blot after different treatments. The expressions of N-Cadherin and Becline-1 were decreased in either LDHA-siRNA+RT or FX-11+RT treatment compared to scr-siRNA+RT or vehicle+RT, while the levels of γH2AX and caspase-3 (active) were increased in either LDHA-siRNA+RT or FX-11+RT treatment compared to scr-siRNA+RT or vehicle+RT. The levels of HIF-1α and Ku70 were significantly reduced after combination of LDHA-siRNA or FX-11 and RT treatment compared to RT plus vehicle treatment. β-actin was used as a loading control. Note: “caspase-3 (a)” indicates caspase-3 (active)”

Our results demonstrated that reduced N-Cadherin expression (EMT marker) was found in PC-3RR cells treated with LDHA-siRNA+RT and FX11+RT compared to the scr-siRNA and vehicle controls (Figure 6) while the suppression of LDHA with single LDHA-siRNA or FX-11 treatments also reduced N-Cadherin expression in PC-3RR cells compared to controls (Figure 6), indicating EMT may be involved in radiosensitization caused by LDHA-siRNA or FX11 inhibition.

To assess the level of hypoxia in LDHA-siRNA or FX11 inhibitor treated cells, the HIF-1α was used as an indicator of hypoxia. We found that HIF-1α was significantly reduced in combination of LDHA-siRNA or FX-11 and RT compared to scr-siRNA+RT or vehicle+RT treated cells (Figure 6), indicating that hypoxia is associated with combination treatment with LDHA-siRNA or FX-11and RT.

Our results also showed that the levels of DNA DSBs marker-γH2AX were significantly increased in LDHA-siRNA, LDHA-siRNA+RT, FX-11, FX-11+RT, treated PC-3RR cells compared to scr-siRNA and scr-siRNA+RT or vehicle controls and vehicle+RT treated PC-3RR cells (Figure 6). Particularly, combination of LDHA-siRNA and RT significantly increased γH2AX level compared to vehicle+RT treatment and no obvious change was found between scr-siRNA or vehicle controls and scr-siRNA+RT or vehicle+RT treated cells (Figure 6), suggesting that there is a synergic effect by combination of LDHA-siRNA and RT in introducing DSBs in PC-3RR cells and improving radiosensitivity.

To evaluate the cellular response to DNA damage after RT, we examined the NHEJ DNA repair pathway protein Ku70 and found the level of Ku70 expression was significantly reduced in combination of LDHA-siRNA or FX-11 and RT compared to scr-siRNA+RT or vehicle+RT treatment (Figure 6), which is consistent with the DSB results and indicates the NHEJ DNA repair pathway plays an important role in combination of LDHA modulation or FX-11 inhibition and RT.

In addition, our results also indicated that single LDHA-siRNA or FX-11 treatment induced more apoptosis (increased Caspase-3-active) in PC-3RR cells compared to scr-siRNA or vehicle control and the combination of LDHA-siRNA/FX-11 and RT enhanced apoptosis to a higher extent (Figure 6). The findings of apoptosis by Western blot were confirmed by immunofluorescence staining (Supplementary Figure S1). Single LDHA-siRNA KD or FX-11 treatment slightly suppressed autophagy (decreased Beclin-1 level) in PC-3RR cells compared to scr-siRNA or vehicle control, and the combination of LDHA-siRNA/FX-11 and RT reduced Beclin-1 to a lower extent compared with LDHA-siRNA or FX-11 treatment alone (Figure 6). These results suggest apoptosis and autophagy mechanisms are associated with LDHA downregulation or FX-11 inhibition mediated CaP radiosensitization. The changes of EMT, hypoxia, DSB, NHEJ, apoptosis, and autophagy pathway markers in PC-3RR cells with different treatments are summarized in Table 1.

**Table 1:** The changes of EMT, hypoxia, DSB, NHEJ, apoptosis, and autophagy pathway markers in PC-3RR cells with different treatments

Pathway/function	LDHA-siRNA vs scr-siRNA	LDHA-siRNA+RT vs LDHA-siRNA	LDHA-siRNA+RT vs scr-siRNA+RT	FX-11 vs vehicle control	FX-11+RT vs FX-11	FX-11+RT vs vehicle control+RT
EMT (N-Cadherin)	−	+	−	−	+	−
Hypoxia (HIF-α)	−	−	−	=	+	−
DSB (γH2AX)	+	=	+	+	+	+
NHEJ (Ku70)	−	−	−	=	−	−
Apoptosis (Caspase-3-active)	+	+	+	+	+	+
Autophagy (Becline-1)	−	−	−	−	−	−

**Notes:** “+” pathway is enhanced; “−” pathways is suppressed; “=” no significant change. The results are from three independent experiments.

## DISCUSSION

In the current study, we firstly developed PC-3RR s.c xenograft tumor model for proteomic biomarker discovery of interesting therapeutic targets. While there was no obvious difference in growth rates between PC-3RR tumors and PC-3 control tumors, the PC-3RR tumors were less responsive to radiation treatment compared to PC-3 control tumors (unpublished observation), suggesting that the RR characteristics were retained in the PC-3RR xenografts *in vivo*. It is well known that angiogenesis, hypoxia, EMT and CSC play very important roles in cancer radioresistance [10]. In PC-3RR tumors, we demonstrated increased angiogenesis (CD31 and VEGFR2) and hypoxia (HIF-1α) as well as enhanced EMT (increased N-Cadherin and reduced E-Cadherin) and elevated CSC markers' expression (CD44 and Oct-4), which is consistent with our previous *in vitro* study in CaP-RR cells [8], further confirming that angiogenesis, hypoxia, EMT and CSC are involved in CaP radioresistance and this model is very suitable for studying CaP radioresistance.

With LC-MS/MS analysis, 378 DEPs were identified between PC-3 and PC-3RR tumor xenografts. PCA data indicated a satisfactory separation of two groups of samples from PC-3 and PC-3RR xenograft tumors. Pathway enrichment analysis could demonstrate 51 pathways to be deregulated in PC-3RR tumors. Among them, 37 pathways are reported to be associated with CaP using Pubmed (http://www.ncbi.nlm.nih.gov/pubmed) database search. Our results indicate that top five pathways associated with CaP radioresistance (ordered according to the number of CaP related publications from Pubmed database) are: VEGF signaling, Integrin signaling, IGF-1 signaling, Glycolysis I and Protein Kinase A signaling. These findings suggest that CaP radioresistance is regulated by a multiple protein network and various important signaling pathways, and that management of these proteins or signaling pathways is promising to develop novel therapies to improve CaP RT.

A number of studies have demonstrated that increased aerobic tumor metabolism (glycolysis) is highly associated with the development of radioresistance by providing a chemically reduced milieu in the tumor microenvironment [11] and inhibition of glycolysis resulted in increased radiosensitivity [12]. The roles of glycolysis in CaP radioresistance are still unclear. Due to the importance of glycolysis in cancer radioresistance and its close link with angiogenesis, hypoxia, EMT and CSC [13-17], this pathway was chosen for further validation. It was found that the key glycolysis pathway proteins-GLUT-1, PKM1/2 and LDHA were increased in PC-3RR xenograft tumors compared to PC-3 tumors, further confirming the activation of glycolysis pathway in CaP radioresistance.

LDHA is a main metabolic enzyme for lactate production which is a terminal product from glycolysis. It is one of the important glycolysis pathway proteins which catalyzes the inter-conversion of pyruvate and lactate. Several lines of evidence indicate that elevated levels of LDHA correlate with a higher grade of aerobic glycolysis and with poor prognosis in cancers [18-21]. It was reported that the high levels of lactate and LDHA are associated with poor response to radiation treatments in head and neck squamous cell carcinoma (HNSCC) [22, 23]. Yamada et al. found that high level of serum LDHA is indicative of poor prognosis in CaP [24]. Koukourakis et al. conducted a study on 83 human CaP biopsies and found that LDH5, an isoform encoded by LDHA gene, is significantly associated with biomedical failure after RT [25], indicating that LDH family is associated with CaP radioresistance. The current study demonstrated that higher levels of LDHA expression were found in CaP-RR cells and PC-3RR xenograft tumors, suggesting that LDHA could be a hallmark in CaP radioresistance, and LDHA down-regulation and inhibition both lead to radiosensitization of CaP-RR cells, which is consistent with the result of LDH5 by Koukourakis et al [25]. The role of LDHA in CaP radioresistance is still unclear. Therefore, a series of functional studies were performed to investigate the mechanisms and pathways that are involved in the LDHA reduction mediated CaP radiosensitization.

ROS has an important implication in cancer radioresistance [9]. As a key event in RT-induced biological processes, it is widely involved in DNA damage, cell cycle arrest and apoptosis [26]. In this study, we found knocking down LDHA using siRNA or treatment with a specific LDHA inhibitor FX-11 combined with RT could increase radiosensitivity in PC-3RR cells accompanied with increased ROS, which is in line with the findings in CaP cell lines treated with LDHA siRNA or LDHA inhibitor by Koukourakis et al [25]. A previous report demonstrated that driving glycolysis and degradation of glucose to lactate supports diminishing of oxidative stress [27]. KD of LDHA was reported to contribute to the increase of mitochondrial ROS, because of the redirection of metabolism away from lactate as the end product, favouring rather the conversion of pyruvate to acetyl-coA and entering the TCA cycle in mitochondria for breast cancer [28]. It was shown that FX-11 treatment induces oxidative stress and cell death in the P493 human lymphoma B cells *in vitro* and inhibited human lymphoma progression [29]. These results suggest that LDHA modulation-induced radiosensitization might partly result from ROS. Therefore, reduction of LDHA-mediated glycolysis in this study could be regarded as a driver of oxygen consumption, which resulted in high level of ROS, and increased ROS in combination treatment can lead to cell death [29].

Our recent study demonstrated that EMT could be a factor leading to the acquisition of CaP radioresistance [8]. Shintani et al. also found that increased EMT is associated with resistance of non-small cell lung cancer (NSCLC) to RT or chemotherapy [30]. Kim et al. reported that microRNA (miR)-34a mediated targeting of EMT increased *in vivo* radiosensitivity in a NSCLC xenograft mouse model [31]. In the current study, we showed that single LDHA-siRNA or FX-11 inhibition resulted in reduction of EMT, and combination treatments reduced EMT compared to single RT, suggesting that reversal of EMT by LDHA modulation could be another possible mechanism for radiosensitizaion in CaP-RR cells.

HIF-1α is stabilized and accumulated under hypoxia. Ample evidence showed that it up-regulates glycolysis genes [32] including LDHA in CaP [33]. An earlier study proved a hypoxia response element was present in the promoter region of the LDHA gene [34]. KD of LDHA and HIF-1α was reported to restore sensitivity to a chemodrug on multiple myeloma cell lines [35]. Hypoxic cells are believed to be more resistant to radiation treatment compared to non-hypoxic cells [36]. When normoxia is dominant rather than hypoxia, activation of various tumor survival and angiogenesis genes is inhibited [29]. Reoxygenation after RT is regarded to improve the outcome of RT [37]. Inhibition of LDHA in human P493 B-lymphoid cells resulted in inhibition of hypoxia [29], indicating LDHA and hypoxia are correlated. In this study, we showed that the HIF-1α was downregulated by LDHA-siRNA combined with RT, suggesting hypoxia could be suppressed by reduced LDHA, thus leading to inhibition of tumor survival genes and promoting radiosensitization.

A critical marker of RT-induced DNA DSBs is the phosphorylation of histone γH2AX [38], which is an indicator of the DSB repair ability after RT [39]. Escalated DSB was observed in PC-3RR cells at both LDHA KD and inhibition of LDHA alone. Combination of LDHA KD or inhibition with RT, further increased DSB in PC-3RR cells compared to RT treatment alone. Furthermore, the DNA DSB repair via NHEJ pathway, as indicated by Ku70 level, was significantly abrogated in LDHA KD, or even depleted in the LDHA KD plus RT treated cells. However, the impaired NHEJ DNA repair mechanism was not obvious in FX-11 treatments as in LDHA KD, suggesting LDHA inhibition by FX-11 does not completely confer DNA repair ability through NHEJ pathway, and the FX-11 treated cells still retain DNA repair ability via other pathways.

Apoptosis has a crucial role in cell death after RT, and autophagy is called as ‘the second apoptosis’. In cancer therapy, the role of autophagy is paradoxical, in which this cellular process may serve as a pro-survival or pro-death mechanism to counteract or mediate the cytotoxic effect of anticancer agents [40]. Le et al. found that silencing or inhibition of LDHA increased apoptosis possibly via induced oxidative stress [29]. As previous described, targeting autophagy was considered as an effective way of overcoming radioresistance [41]. Meng et al. demonstrated that silencing PKM2 in the glycolysis pathway was shown to sensitize RT in NSCLC via inhibiting autophagy [12]. It was also reported that inhibition of autophagy by rapamycin and apoptosis inducer could effectively sensitize a lung cancer cell line to radiation [42]. In this study, we found that combination of LDHA-siRNA or FX-11 inhibitor with RT both markedly increased apoptosis and reduced autophagy level, suggesting that both apoptosis and autophagy are involved in CaP radiosensitization effect.

The functional studies have proved that LDHA KD or inhibition can lead to the changes of ROS, EMT, hypoxia, DSB, apoptosis and autophagy, thus resulting in the sensitization of CaP RR cells. Because of its tight linkage to different pathways, LDHA is revealed to be not only a hallmark of CaP radioresistance but also a favorable target for radiosensitization treatment of the CaP-RR patients.

In summary, in this study, we have identified a list of potential RR markers and important signaling pathways from PC-3RR xenograft mouse model, and found that LDHA is a potential therapeutic target associated with CaP radioresistance. In addition, we demonstrate that the radiosensitization effect of combination of interfering LDHA with RT is associated with enhanced ROS, apoptosis and DSBs as well as reduced EMT and autophagy. Our findings suggest that LDHA is an ideal therapeutic target to develop combination therapy to overcome CaP radioresistance.

## MATERIALS AND METHODS

### Cell lines and cell culture

PC-3, DU145, LNCaP cell lines were obtained from American Type Culture Collection (ATCC) (Rockville, MD, USA). PC-3RR, DU145RR, and LNCaPRR cell lines were developed and the confirmed in our previous study [8]. All cell lines used were cultured in RPMI-1640 medium supplemented with 10% heated-inactivated fetal bovine serum (FBS), 50 U/mL of penicillin and 50 μg/mL of streptomycin. All cell lines were maintained in a humidified incubator at 37°C and 5% CO_2_. The identities of all cell lines were confirmed by short tandem repeat profiling. All cell lines were regularly tested to confirm the absence of mycoplasma contamination using the LookOut^®^ mycoplasma PCR detection kit (Sigma-Aldrich Pty Ltd, NSW, Australia).

### Antibodies

Antibodies were obtained from different sources. The detailed information and conditions for all antibodies are listed in Table 2.

**Table 2:** Antibodies used for western blot (WB), immunofluorescence (IF) and immunohistochemistry (IHC) staining

Antibody	Source	Type	Dilution	Incubation time	Temperature	Application
Rabbit anti-E-Cadherin antibody	Abcam	PAb	1:100	O/N	4°C	IHC
Rabbit anti-human N-Cadherin antibody	Abcam	PAb	1:500	O/N	4°C	IHC, WB
Rabbit anti-human Ku70 antibody	Abcam	PAb	1:1000	O/N	4°C	WB
Rabbit anti-human γH2AX antibody	Abcam	PAb	1:1000	O/N	4oC	WB
Rabbit anti-human active Caspase-3 antibody	Abcam	PAb	1:500	O/N	4°C	WB
Rabbit anti-Becline-1 antibody	Cell signalling Technology	PAb	1:1000 (WB)	O/N	4°C	WB
Rabbit anti-human Becline-1 antibody	Cell signalling Technology	PAb	1:1000	O/N	4°C	WB
Mouse anti-human Beta-actin antibody	Sigma Altrich	MAb	1:5000	O/N	4°C	WB
Rat anti-mouse CD31 antibody	BD pharmingen	MAb	1:100	O/N	4°C	IHC
Rabbit anti-VEGFR2 antibody	Abcam	PAb	1:100	O/N	4°C	IHC
Mouse anti-CD44 antibody	Santa Cruz Biotechnology	MAb	1:200	O/N	4°C	IHC
Rabbit anti-Oct4 antibody	Abcam	PAb	1:500	O/N	4°C	IHC
Mouse anti-human HIF-1α antibody	Abcam	MAb	1:500 (WB) 1:100 (IHC)	O/N	4°C	IHC, WB
Rabbit anti-human PKM1/2 antibody	Cell signalling	PAb	1:1000 (WB) 1:100 (IHC)	O/N	4°C	IHC, WB
Rabbit anti-human GLUT-1 antibody	Abcam	PAb	1:1000 (WB) 1:100 (IHC)	O/N	4°C	IHC, WB
Rabbit anti-human LDHA antibody	Abcam	PAb	1:2000 (WB) 1:400 (IHC)	O/N	4°C	IHC, WB
Goat anti-rabbit Alexa Fluor® 488 Dye Conjugate	Invitrogen	IgG	1:1000 (IF)	60 mins	rt	IF
Goat anti-rabbit IgG-HRP	Santa Cruz Biotechnology	PAb	1:2000	45 mins	rt	WB
Goat anti-mouse IgG-HRP	Santa Cruz Biotechnology	PAb	1:2000	45 mins	rt	WB
Rabbit anti-rat immunoglobulins, biotinlyated	Dako Pty. Ltd.	PAb	1:100	45 mins	rt	IHC
Goat anti-rabbit IgG-HRP	Dako Pty. Ltd.	PAb	1:100	45 mins	rt	IHC
Rabbit anti-mouse IgG-HRP	Dako Pty. Ltd.	PAb	1:100	45 mins	rt	IHC
Mouse anti-human IgG1-negative	Dako Pty. Ltd.	IgG	1:100	O/N	4°C	IHC
Rabbit anti-human IgG1-negative	Dako Pty. Ltd.	IgG	1:100	O/N	4°C	IHC

**Notes:** HRP: horseradish peroxide;IF: immunofluorescence;IHC: immunohistochemistry; MAb: monoclonal antibody; O/N: overnight; PAb: polyclonal antibody; rt: room temperature; WB: Western Blot.

### Establishment of PC-3 and PC-3RR animal xenograft models *in vivo*

Seven weeks male NOD/SCID mice (Animal Resources Centre, Western Australia) were housed under specific pathogen-free conditions in facilities approved by the University of New South Wales (UNSW) Animal Care and Ethics Committee (ACEC) (ACEC 13/118B). Mice were kept at least 1 week before experimental manipulation. All mice remained healthy and active during the experiments. The CaP s.c model was established following our published method [43]. Briefly, cultured PC-3 and PC-3RR cells (2 × 10^6^/injection) in 100 uL Dulbecco's Phosphate-Buffered Saline (DPBS) were implanted subcutaneously in the right rear flank region of mice (n=5 mice/per group). Tumor progression was documented weekly by measuring tumor sizes using callipers. Tumor volumes were calculated as follows: length x width x height x 0.52 (in millimetres) [44].

For growth rate study, the mice were euthanized 7 weeks post cell inoculation. For each tumor, half were snap-frozen using liquid nitrogen for proteomics study and the rest separated for paraffin embedding and frozen sectioning for histological examination and IHC.

### Preparation of protein samples for LC-MS/MS

Frozen PC-3 or PC-3RR xenograft tissues were minced with stainless steel scalpels. The mince tissues were then ground in liquid nitrogen in a mortar using a pestle. The tissue powder was resuspended using lysis buffer (50 mmol/L Tris-HCl (pH 8.0), 150 mmol/L NaCl, 0.1% sodium dodecyl sulfate (SDS), 10 mmol/L NaF, 1 mmol/L Na_3_VO_4_, 0.5% sodium deoxycholate and 1% Triton X-100) at a ratio of 1 mg tissue:5 mL buffer, followed by a bead beating method. Briefly, 0.13g zirconium beads (0.1 mm diameter)/100 μL of lysis buffer were added into mixture, and bead beating was performed on the highest setting for 80 seconds and returned to ice for 5 mins. Bead beating was repeated 4 times. Solution was centrifuged at 30000g for 40 min and the supernatant was collected. Protein concentration was determined by the BCA protein assay kit (Pierce, USA). Protein samples were digested according to a published method [45]. A total of 100 μg of total cell lysate from each sample was precipitated in 100% ice-cold acetone at 1:4 (v/v) protein solution:acetone dilution. Trypsin (in 50 mM ammonium bicarbonate buffer, pH 8.5) was added at an enzyme/substrate ratio of 1:100 (w/w) and the samples were incubated overnight (o/n) to allow for digestion. The lyophilized samples were eluted by using C18 stage tips and collected in mass spectrometry tubes.

### LC-MS/MS

LC-MS/MS analysis was carried out for PC-3 and PC-3RR xenograft samples using an LTQ Orbitrap Velos ETD (Thermo Scientific, US) at Bioanalytical Mass Spectrometry Facility (BMSF), UNSW. Digested peptides were reconstituted in 10 μL 0.1% formic acid and separated by nano-LC using an Ultimate 3000 HPLC and autosampler (Dionex, Amsterdam, Netherlands). The sample (0.2 μL) was loaded onto a micro C18 pre-column (300μm×5mm, Dionex, Scoresby, VIC, Australia) with Buffer A (98% H_2_O, 2% CH_3_CN, 0.1% TFA) at 10 μL/min. After washing, the pre-column was switched (Valco 10 port valve, Dionex) into line with a fritless nano column (75 μm i.d × 15cm) containing reverse phase C18 media (3 μm, 200 Å Magic, Michrom Bioresoures). Peptides were eluted using a linear gradient of Buffer A to Buffer B (98% CH_3_CH, 2% H_2_O, 0.1% formic acid) at 0.25 μL/min over 60 min. High voltage (2000V) was applied to low volume tee (Upchurch Scientific, Oak Harbor, WA, USA) and the column tip positioned 0.5 cm from the heated capillary (*T*=280°C) of an Orbitrap Velos (Thermo Electron, Bremen, Germany) mass spectrometer. Positive ions were generated by electrospray and the Orbitrap was operated in a data-dependent acquisition (DDA) mode. A survey scan 350-1750 *m/z* was acquired in the Orbitrap (Resolution=30000 at 400 *m/z*, with an accumulation target value of 1000000 ions) with lockmass enabled. Up to the 10 most abundant ions (>5000 counts) with charge states +2 to +4 were sequentially isolated and fragmented within the linear ion trap using collisionally induced dissociation with an activation *q*=0.25 and activation time of 30 ms at a target value of 30000 ions. The *m/z* ratios selected for MS/MS were dynamically excluded for 30s [46].

### Progenesis QI analysis

MS peak intensities were analyzed using Progenesis QI data analysis software v4 (Waters). Ion intensity maps from each run were aligned to a reference sample and ion feature matching was achieved by aligning consistent ion *m/z* and retention times. The peptide intensities were normalized against total intensity (sample specific log-scale abundance ratio scaling factor) and compared between groups by one-way analysis of variance (ANOVA, *p*≤0.05 for statistical significance) and *post hoc* multiple comparison procedures. Type I errors were controlled by False Discovery Rate (FDR) with *q* value significance set at 0.01 [47]. Results are reported as mean ± SD (normalized ion intensity score).

### Protein dataset

Peak lists of proteins were generated using Mascot Daemon/extract_msn (Matrix Science, Thermo, London, UK) using the default parameters, and submitted to Mascot 2.1 (Matrix Science). All MS/MS spectra of differentiating peptides were searched against human non-redundant NCBInr database using the Mascot search program (Matrix Science, London, UK, www.matrixscience.com) for protein identification with the following criteria: (1) species, Homo sapiens; (2) allowed one missed cleavage; (3) variable modifications, Oxidation (M), Phospho (ST) and Phospho (Y); (4) peptide tolerance, ±6 ppm; (5) MS/MS tolerance, ± 0.6Da; (6) peptide +2, +3 and +4; and (7) enzyme specificity, none. The results were imported into Progenesis QI LC-MS software and peptides were considered to be confidently identified when matches had a high ion score >20 and peptides were assigned to a protein.

### Ingenuity

IPA (Ingenuity^®^ Systems, www.ingenuity.com) was used to assess pathways of differentially regulated proteins. The accession numbers of DEPs, average normalized abundances, fold changes and *p*-values were submitted to IPA. The protein pathways and the terms were identified through IPA database search.

### Immunohistochemistry

Paraffin sections of xenograft tumors were used for IHC staining of angiogenesis, EMT, CSC and glycolysis markers as well as for H&E staining (tumor histology). All staining was performed following our previously published methods [48, 49]. Briefly, paraffin sections were de-paraffinized and rehydrated, then incubated with primary antibodies o/n at 4 °C. Slides were then incubated with rabbit anti-mouse or goat anti-rabbit IgG/HRP second antibody (1:100 dilution) for 45 min. Frozen 5μM sections of xenograft tumors were used for CD31 and CD44 staining to examine the micro-vessel density (MVD) and CSC. Sections were incubated with rat anti-mouse CD31 or mouse anti-human CD44 monoclonal antibody (MAb) (1:100 dilution) o/n at 4°C then incubated with rabbit anti-rat HRP or Goat anti-mouse HRP (1:100 dilution) for 45 min at room temperature. All sections were finally developed with 3,3′ diaminobenzidine (DAB) substrate solution (Sigma-Aldrich, Pty Ltd, Castle Hills, NSW, Australia), then counterstained with hematoxylin (Thermo Fisher Pty Ltd, VIC, Australia). Control slides were treated in an identical manner, by using isotype antibodies or omitting primary antibody as a negative control.

### RNA extraction and qRT-PCR

Approximately 1mg of tumors from PC-3 and PC-3RR xenografts was collected and ground in liquid nitrogen. Immediately after liquid nitrogen evaporated, the total RNA was extracted and purified using the RNeasy Plus Mini Kit (Qiagen, VIC, Australia) according to the manufacturer's instructions. The concentrations of total RNA from tissues were measured by a ND-2000 NanoDrop spectrophotometer (NanoDrop Technologies, Wilmington, DE). Two micrograms (mg) of total RNA from each sample were reverse transcribed to cDNA using the SuperScript III First-strand Synthesis System Kit (Invitrogen Pty Ltd, VIC, Australia), according to the manufacturers protocol. All mRNA expression of the LDHA and β-actin gene was assessed using qRT-PCR. A Rotor-Gene instrument (Corbett Life Science, Sydney, Australia) was used for automated qRT-PCR setup of the reactions. After three independent experiments, the REST 2009 V2.0.13 (Qiagen, VIC, Australia) software was used for calculation and analysis of LDHA gene expression.

### LDHA siRNA transfection

Silencer Select LDHA siRNAs and Negative Control scramble scr-siRNA were purchased from Invitrogen (Invitrogen Australia Pty Ltd, Melbourne, VIC, Australia). Transfection was performed by incubating the cells with LDHA-siRNA or scr-siRNA and LipofectAMINE 2000 at 37°C for 1-7 days, following the manufacturer's protocol. For all functional studies, LDHA-siRNA was used for 4 days to achieve the best KD effect and maintain a healthy status of the cells.

### Cytotoxicity of FX-11

FX-11 (a LDHA inhibitor) was purchased from Merck Millipore (VIC, Australia) and dissolved in DMSO. Cytotoxicity of FX-11 was evaluated on PC-3RR cells using an MTT assay following a published method [48]. Briefly, 2000 cells were seeded in 96-well plates incubated in culture media for 24 h. Cells were then treated with a range of concentrations of FX-11 (0–1 mM) or the same volume of DMSO as the vehicle control in fresh media for 48 h. The absorbance (OD) was read at 560 nm on a BIO-TEC micro-plate reader (BIO-RAD, Hercules, CA, USA). The IC_20_ value (20% inhibitory concentration) of FX-11 was calculated and chosen for the following experiments.

### Colony formation assay

PC-3RR cells with different treatments including scr-siRNA control, LDHA-siRNA, DMSO vehicle control and FX-11 were seeded for a clonogenic survival assay following exposure to different dose of radiations 0, 2, 4, 6, and 8 Gy. Different numbers of cells were seeded in 6 well plates in triplicates: 500 cells for 0 Gy, 1000 cells for 2 Gy, 2000 cells for 4 Gy, 4000 cells for 6 Gy, and 8000 cells for 8 Gy. After irradiation, the cells were cultured for 14 days and the number of surviving colonies (defined as a colony with >50 cells) were counted. Data from radiation-treated cells were normalized against the untreated cells (scored as 100% colony forming ability). Plating efficiencies and survival fractions were calculated to obtain survival parameters and plot cell survival curves.

### ROS assay

Intracellular ROS was measured by flow cytometry. The semi-confluent cells (0.5~1 × 10^6^) in 25cm^2^ flasks were treated with scr-siRNA, LDHA-siRNA, DMOS vehicle and FX-11, after 90 hours of scr-siRNA and LDHA-siRNA treatments or 42 hours of DMSO vehicle and FX-11 treatments, the cells were irradiated at 6 Gy. At 6 hours post RT, culture media was replaced with CellROX Green Reagent (Molecular Probes; MA USA) solution (1:1000 in plain RPMI-1640) and was incubated for 20 min. Following detachment and washing, cells were analyzed using a FACS Canto II Flow Cytometer (Becton, Dickinson and Company, BD Biosciences, San Jose, USA).

### Western blot

Cultured PC-3RR cells in 25 cm^2^ flasks were treated with scr-siRNA, scr-siRNA+RT, LDHA-siRNA, LDHA-siRNA+RT, vehicle control, vehicle control+RT, FX-11, FX-11+RT. There was a 90 hours interval between LDHA-siRNA/scr-siRNA and RT treatments or a 42 hours interval between FX-11/vehicle control and RT treatments. At 6 hours post RT treatments, proteins were extracted. Protein expression levels were determined by Western blot analysis as described [48]. Briefly, whole cell lysates were separated by NuPAGE Novex 4–12% Bis-Tris gel electrophoresis and then transferred to polyvinylidene difluoride membrane. After blocking non-specific sites with 5% bovine serum albumin, the membrane was incubated with different primary antibodies (see Table 2), followed by incubation with HRP-conjugated secondary antibodies (goat anti-mouse or goat anti-rabbit appropriate for the host species of the primary antibody) (1:2000 dilution). Immunoreactive bands were detected using enhanced chemiluminescence (ECL) substrate (Pierce Chemical Co, Rockford, USA), and imaged using the ImageQuant LAS4000 system (GE Health care, USA). To confirm equal loading of protein lysates, membranes were re-probed using mouse anti-human β-actin antibody (1:5000 dilution), then processed as above.

### Immunofluorescent staining

Immunofluorescence staining was performed as previously described [8]. Briefly, the cultured PC-3RR cells with different treatments as mentioned above in Western blotting were cytospined at room temperature and then incubated with 10% normal goat serum in TBS for 20 min to suppress the nonspecific binding of IgG. The cells were then incubated with anti-caspase-3-active (1:500) o/n at 4 °C. After rinsing in TBS, cells were incubated for 45 min in goat anti-rabbit Alexa Fluor-488 conjugate secondary antibodies (1:1000 dilutions) at room temperature. Propidium iodide (PI) (0.2 mg/L) was used to stain the nuclei. Negative controls were treated identically but omitted with the primary antibodies. Immunofluorescence was visualized using an FV 300/FV500 Olympus laser scanning confocal microscope (Olympus).

### Detection of apoptosis using acridineorange/ethidium bromide (AO/EB) staining

The cultured PC-3RR cells with different treatments as mentioned above in Western blot were prepared for detection of apoptosis by AO/EB staining using our published method [50].

### Statistical analysis

ANOVA was used in progenesis QI analysis. All experiments were performed at least three times and data was presented as the mean±standard deviation (SD), unless otherwise indicated. Data from different groups were compared using the two-tail student's t test. All *p* values were two-sided. *p*<0.05 was considered significant. The statistical analysis of immunostaining intensity in animal xenografts was performed as described in our previous publication [51]. All numerical statistical analyses were performed using the GraphPad Prism 6 package (GraphPad, CA, USA).

## SUPPLEMENTARY MATERIALS FIGURE AND TABLES






