# Supplementary material for: Proteomic identification of the lactate dehydrogenase A in a radioresistant prostate cancer xenograft mouse model for improving radiotherapy
Source: Oncotarget. 2016 Sep 30;7(45):74269–85. doi: 10.18632/oncotarget.12368 (PMC5342052; doi:10.18632/oncotarget.12368)
Supplement: Supplementary file 1 [file oncotarget-07-74269-s001.pdf]

Proteomic identification of the lactate dehydrogenase A in a radioresistant prostate cancer xenograft mouse model for improving radiotherapy

SUPPLEMENTARY FIGURE AND TABLES

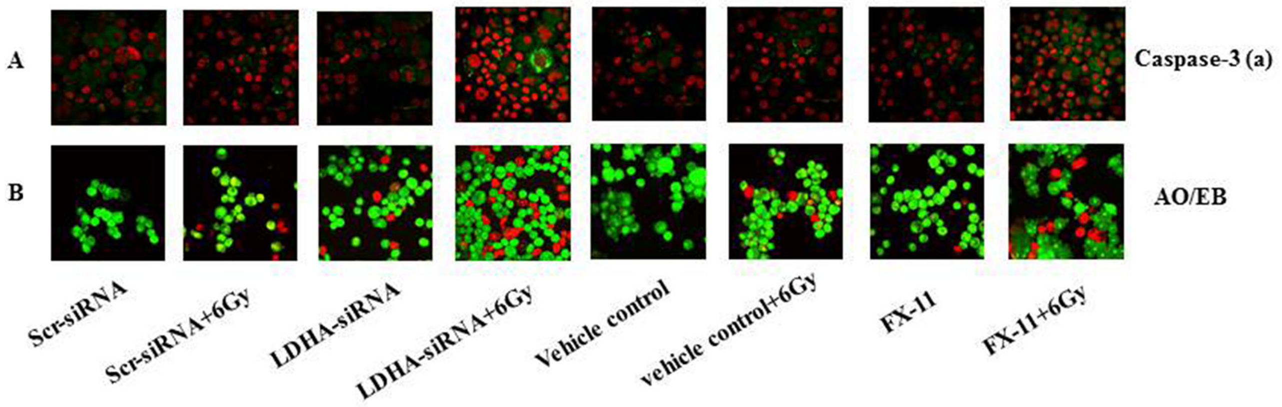

Supplementary Figure S1: Confirmation of apoptosis after different treatments by fluorescence staining. Expression of Caspase-3 (active) (apoptosis) and cell morphology were examined after different treatments by immunofluorescence staining. **A.** The staining intensity of Caspase-3 (active) was up-regulated in combination of LDHA KD or inhibitor and RT treated cells compared to single treatments or vehicle treatments. **B.** The apoptotic cells were also visualized by AO/EB staining. In Caspase-3 (active) study, green indicates viable cells while red indicates non-viable cells. In AO/EB experiment, condensed and fragmented nuclear chromatin characteristic of apoptosis is clearly seen in treated cells using acridine orange/ethidium bromide staining and confocal microscopy (red) while control-treated cells appeared normal (green). Magnification x 600 in all images.

Supplementary Table S1: Intensity of expression of vasculature, hypoxia, EMT, CSC and glycolysis markers in PC3 and PC3-RR s.c. xenografts by IHC

Name of xenograft	IHC intensity									
	CD31	VEGFR2	HIF-1α	N-Cadherin	E-Cadherin	CD44	Oct4	GLUT-1	PKM1/2	LDHA
PC3	0~1	0~1	0~1	0~1	1~2	0~1	0~1	1~2	0~1	0~1
PC3-RR	1~2	2~3	1~2	2~3	0~1	1~2	2~3	2~3	1~2	2~3

Notes: IHC staining scores: 0=negative; 1=weak; 2=moderate; 3=strong.

Supplementary Table S2: Significantly differentially expressed proteins between PC-3 and PC-3RR xenografts.

See Supplementary File 1

Supplementary Table S3: Significant signaling pathways associated with CaP radioresistance identified by Ingenuity Pathways Analysis.

See Supplementary File 2
