# Supplementary material for: Proteomic identification of the lactate dehydrogenase A in a radioresistant prostate cancer xenograft mouse model for improving radiotherapy
Source: Oncotarget. 2016 Sep 30;7(45):74269–85. doi: 10.18632/oncotarget.12368 (PMC5342052; doi:10.18632/oncotarget.12368)
Supplement: Supplementary file 2 [file oncotarget-07-74269-s002.doc]

**Table S2. Significantly differentially expressed proteins between PC-3 and PC-3RR xenografts**

| Accession | Peptide | Score | Anova (p)* | Fold | Description | Average Normalised Abundances | |
| --- | --- | --- | --- | --- | --- | --- | --- |
|  |  |  |  |  |  | Naive | RR |
| gi|5454154 | 3 (1) | 79.3 | 6.37E-03 | 221.05 | peptidyl-prolyl cis-trans isomerase H [homo sapiens] | 1.3 | 286.7 |
| gi|10190712 | 2 (1) | 142.38 | 3.57E-06 | 53.25 | protein S100-A14 [homo sapiens] | 1582.2 | 29.71 |
| gi|12804225 | 6 | 216.36 | 0.03 | 11.09 | Unknown (protein for IMAGE:3543711), partial [homo sapiens] | 6867.97 | 7.62E+04 |
| gi|16307067 | 4 (3) | 124.25 | 5.84E-04 | 9.63 | SUB1 homolog (S. cerevisiae) [homo sapiens] | 292.76 | 2819.95 |
| gi|30311 | 3 (1) | 98.89 | 0.02 | 7.04 | cytokeratin 18 (424 AA) [homo sapiens] | 349.95 | 49.7 |
| gi|187302 | 17 (1) | 1047.79 | 0.01 | 6.68 | epithelial cell marker protein 1 [homo sapiens] | 262.07 | 39.25 |
| gi|231250 | 6 (1) | 204.18 | 0.05 | 5.86 | Chain A, The Nadph Binding Site On Beef Liver Catalase | 1306.31 | 7650.71 |
| gi|7023464 | 2 (1) | 52.19 | 1.13E-06 | 4.38 | unnamed protein product [homo sapiens] | 4844.14 | 1107.21 |
| gi|2160517 | 4 (3) | 194.94 | 2.01E-07 | 4.3 | ladinin [homo sapiens] | 1.79E+04 | 4148.33 |
| gi|4826774 | 5 | 355.05 | 2.81E-08 | 3.97 | ubiquitin-like protein ISG15 precursor [homo sapiens] | 4.12E+05 | 1.04E+05 |
| gi|2624886 | 2 | 118.55 | 2.16E-03 | 3.92 | Chain A, Aldehyde Dehydrogenase From Bovine Mitochondria | 1523.96 | 5978.15 |
| gi|6912286 | 5 (4) | 310.55 | 1.23E-04 | 3.6 | caspase-14 precursor [homo sapiens] | 4.15E+04 | 1.15E+04 |
| gi|6009628 | 4 (3) | 122.55 | 0.01 | 3.33 | brain carboxylesterase hBr3 [homo sapiens] | 1717.55 | 5717.29 |
| gi|28678 | 2 | 109.93 | 5.23E-05 | 3.28 | unnamed protein product [homo sapiens] | 343.94 | 1129.49 |
| gi|245850 | 2 | 116.57 | 4.74E-04 | 2.98 | S-100P=Ca(2+)-binding protein [human, placenta, Peptide, 91 aa] | 2.18E+04 | 7307.99 |
| gi|158937236 | 4 (3) | 146.42 | 7.66E-03 | 2.68 | puromycin-sensitive aminopeptidase [homo sapiens] | 3415.76 | 9151.02 |
| gi|307109 | 2 | 61.54 | 6.01E-04 | 2.62 | lysosomal membrane glycoprotein-1 [homo sapiens] | 2915.66 | 1114.62 |
| gi|6841256 | 2 (1) | 54.79 | 6.93E-03 | 2.6 | HSPC303 [homo sapiens] | 2012.24 | 774.45 |
| gi|34529624 | 2 (1) | 57.45 | 1.52E-03 | 2.57 | unnamed protein product [homo sapiens] | 376.31 | 967.86 |
| gi|3641398 | 14 (3) | 601.77 | 3.33E-06 | 2.5 | NADP-dependent isocitrate dehydrogenase [homo sapiens] | 6.31E+04 | 2.53E+04 |
| gi|119579011 | 2 (1) | 91.66 | 0.02 | 2.44 | hCG1647467 [homo sapiens] | 819.68 | 2003.82 |
| gi|577295 | 3 (2) | 154.34 | 2.84E-05 | 2.42 | KIAA0088 [homo sapiens] | 4107.13 | 9959.25 |
| gi|20151189 | 13 (2) | 693.52 | 1.39E-05 | 2.4 | Chain A, Structure Of Human Glutamate Dehydrogenase-Apo Form | 2.11E+04 | 5.05E+04 |
| gi|4503971 | 2 | 69.64 | 7.47E-04 | 2.37 | rab GDP dissociation inhibitor alpha [homo sapiens] | 4029.49 | 9548.6 |
| gi|35830 | 3 (1) | 187.33 | 6.75E-03 | 2.29 | ubiquitin activating enzyme E1 [homo sapiens] | 659.25 | 1507.08 |
| gi|558528 | 5 (1) | 312.42 | 0.02 | 2.28 | proteasome subunit Y [homo sapiens] | 2317.19 | 1015.66 |
| gi|1942990 | 6 (3) | 404.2 | 0.03 | 2.18 | Chain E, Structure Of Bovine Heart Cytochrome C Oxidase At The Fully Oxidized State | 3.74E+04 | 8.16E+04 |
| gi|4757908 | 3 (1) | 171.63 | 4.21E-03 | 2.15 | calcyphosin isoform a [homo sapiens] | 3053.64 | 1423.12 |
| gi|528281421 | 9 (6) | 579.43 | 3.81E-04 | 2.14 | band 4.1-like protein 3 isoform 3 [homo sapiens] | 2.85E+04 | 6.12E+04 |
| gi|4503483 | 12 | 475.56 | 4.93E-04 | 2.14 | elongation factor 2 [homo sapiens] | 5.28E+04 | 1.13E+05 |
| gi|4757756 | 14 (1) | 768.98 | 0.01 | 2.11 | annexin A2 isoform 2 [homo sapiens] | 4240.58 | 8948.39 |
| gi|32189394 | 19 (1) | 1565.05 | 2.14E-03 | 2.09 | ATP synthase subunit beta, mitochondrial precursor [homo sapiens] | 7515.38 | 3602.82 |
| gi|5174661 | 4 | 134.2 | 3.40E-05 | 2.08 | protein S100-A2 [homo sapiens] | 5.72E+04 | 2.75E+04 |
| gi|4579909 | 2 | 50.98 | 3.27E-04 | 2.05 | apg-2 [homo sapiens] | 4756.6 | 9763.21 |
| gi|31608 | 11 (10) | 568.4 | 5.07E-08 | 2.03 | glucan 1, 4-alpha-glucosidase [homo sapiens] | 3.78E+05 | 1.86E+05 |
| gi|291856 | 3 (1) | 115 | 1.49E-03 | 2.03 | amyloid protein [homo sapiens] | 1557.25 | 765.59 |
| gi|157057089 | 3 | 105.54 | 1.23E-03 | 2.03 | mucin 5AC, oligomeric mucus/gel-forming [homo sapiens] | 8516.89 | 4194.54 |
| gi|29470 | 2 | 95.97 | 3.19E-07 | 2.03 | Human basement membrane heparan sulfate proteoglycan core protein [homo sapiens] | 1.76E+04 | 8669.57 |
| gi|5031573 | 2 | 63.41 | 2.29E-05 | 2.03 | actin-related protein 3 isoform 1 [homo sapiens] | 1.48E+04 | 3.02E+04 |
| gi|14210480 | 3 (2) | 134.08 | 1.14E-05 | 1.99 | cAMP-dependent protein kinase inhibitor beta isoform 1 [homo sapiens] | 1.45E+04 | 7280.74 |
| gi|183182 | 2 (1) | 109.98 | 3.29E-04 | 1.97 | guanine nucleotide-binding regulatory protein alpha-inhibitory subunit [homo sapiens] | 4834.92 | 9513.35 |
| gi|42543459 | 5 (1) | 237.43 | 7.79E-04 | 1.93 | Chain A, Crystal Structure Of A Mu-Like Calpain | 829.3 | 1600.51 |
| gi|4504165 | 12 (3) | 605.22 | 1.42E-05 | 1.91 | gelsolin isoform a precursor [homo sapiens] | 1.57E+04 | 8202.37 |
| gi|6694937 | 4 | 228.78 | 2.35E-04 | 1.89 | nudix hydrolase NUDT5 [homo sapiens] | 6.05E+04 | 3.20E+04 |
| gi|565169 | 2 (1) | 149.93 | 0.04 | 1.88 | early-pregnancy factor, EPF=chaperonin 10 homolog [human, platelets, Peptide Partial, 27 aa, segment 2 of 2] | 2986.88 | 5604.24 |
| gi|13491174 | 11 (10) | 477.94 | 5.27E-05 | 1.87 | MARCKS-related protein [homo sapiens] | 4.36E+05 | 2.33E+05 |
| gi|4506661 | 3 | 139.35 | 1.21E-04 | 1.86 | 60S ribosomal protein L7a [homo sapiens] | 3.36E+04 | 1.81E+04 |
| gi|1374792 | 4 | 210.32 | 2.24E-04 | 1.82 | selenium-binding protein [homo sapiens] | 4.59E+04 | 2.53E+04 |
| gi|4503117 | 3 | 186.21 | 3.96E-06 | 1.81 | cystatin-B [homo sapiens] | 1.18E+05 | 6.51E+04 |
| gi|11034825 | 5 (2) | 258.12 | 5.28E-04 | 1.8 | methionine adenosyltransferase 2 subunit beta isoform 1 [homo sapiens] | 5285.15 | 9530.5 |
| gi|6453380 | 6 (3) | 213.61 | 0.03 | 1.8 | integrin beta 4 [homo sapiens] | 1.38E+04 | 7656.14 |
| gi|2781202 | 3 (1) | 141.95 | 0.02 | 1.79 | Chain A, Three-Dimensional Structure Of Human Electron Transfer Flavoprotein To 2.1 A Resolution | 3831.57 | 2143.1 |
| gi|6715568 | 2 (1) | 72.02 | 3.76E-03 | 1.79 | serine/threonine-protein phosphatase 2B catalytic subunit alpha isoform isoform 1 [homo sapiens] | 4181.26 | 7470.74 |
| gi|5802966 | 6 (3) | 233.91 | 1.21E-05 | 1.76 | destrin isoform a [homo sapiens] | 3.95E+04 | 6.96E+04 |
| gi|14424542 | 2 | 149.9 | 6.08E-04 | 1.76 | RPL14 protein [homo sapiens] | 1.04E+04 | 5901.38 |
| gi|10434838 | 2 (1) | 64.05 | 0.02 | 1.76 | unnamed protein product [homo sapiens] | 724.08 | 412.1 |
| gi|189054911 | 13 (12) | 696.46 | 4.30E-09 | 1.75 | unnamed protein product [homo sapiens] | 5.08E+05 | 2.90E+05 |
| gi|5031857 | 11 (3) | 459.95 | 0.02 | 1.75 | L-lactate dehydrogenase A chain isoform 1 [homo sapiens] | 2.06E+04 | 3.60E+04 |
| gi|5032057 | 5 (3) | 330.62 | 3.80E-03 | 1.75 | protein S100-A11 [homo sapiens] | 1.15E+05 | 6.56E+04 |
| gi|62420916 | 2 (1) | 153.91 | 0.05 | 1.75 | actin-like protein [homo sapiens] | 2454.01 | 4302.1 |
| gi|5729997 | 2 | 79 | 2.08E-03 | 1.74 | ras-related protein Rab-27B [homo sapiens] | 6641.13 | 3818.53 |
| gi|6272557 | 3 | 151.21 | 0.04 | 1.73 | ERO1L [homo sapiens] | 1.11E+04 | 1.92E+04 |
| gi|6912494 | 4 (2) | 149.57 | 5.30E-04 | 1.73 | microtubule-associated protein RP/EB family member 1 [homo sapiens] | 4395.37 | 7625.22 |
| gi|131804 | 3 (1) | 142.55 | 0.01 | 1.73 | RecName: Full=Ras-related protein Rab-10 | 1041.54 | 1804.75 |
| gi|179832 | 5 | 276.24 | 1.51E-04 | 1.72 | calnexin [homo sapiens] | 3.95E+04 | 6.80E+04 |
| gi|453369 | 4 | 189.45 | 5.93E-06 | 1.71 | maspin [homo sapiens] | 2.01E+04 | 1.18E+04 |
| gi|5803092 | 4 | 108.93 | 3.58E-04 | 1.7 | methionine aminopeptidase 2 [homo sapiens] | 1.01E+04 | 5927.29 |
| gi|39930375 | 3 (1) | 91.76 | 5.99E-03 | 1.69 | protein enabled homolog isoform b [homo sapiens] | 7447.03 | 4400.5 |
| gi|68085578 | 23 (1) | 1424.93 | 0.01 | 1.68 | Tyrosine 3-monooxygenase/tryptophan 5-monooxygenase activation protein, zeta polypeptide [homo sapiens] | 1291.31 | 770.14 |
| gi|6563288 | 5 (1) | 332.73 | 1.46E-03 | 1.68 | ubiquitin-like product Chap1/Dsk2 [homo sapiens] | 9405.16 | 5592.83 |
| gi|1136741 | 7 (6) | 370.15 | 1.78E-04 | 1.66 | KIAA0002 [homo sapiens] | 2.26E+04 | 3.73E+04 |
| gi|1181996 | 5 (2) | 248.12 | 0.02 | 1.66 | hair type II basic keratin [homo sapiens] | 6555.65 | 1.09E+04 |
| gi|4885257 | 2 | 96.68 | 1.61E-03 | 1.66 | GTP cyclohydrolase 1 feedback regulatory protein [homo sapiens] | 1.06E+04 | 6382.83 |
| gi|50417352 | 11 (6) | 641.39 | 9.94E-04 | 1.65 | DPYSL3 protein [homo sapiens] | 5.46E+04 | 8.99E+04 |
| gi|1488414 | 10 (2) | 560.95 | 9.53E-04 | 1.65 | N8 gene product long isoform, N8L protein=D52 homolog/leucine zipper protein {alternatively spliced, clone HK4a1} [human, normal kidney, Peptide, 248 aa] | 1.77E+05 | 1.07E+05 |
| gi|1203982 | 5 (4) | 373.66 | 8.21E-04 | 1.65 | NAD+-dependent 15-hydroxyprostaglandin dehydrogenase [homo sapiens] | 3.59E+04 | 5.93E+04 |
| gi|535032 | 3 (2) | 123.51 | 7.59E-03 | 1.65 | peroxisomal acyl-CoA oxidase [homo sapiens] | 5780.06 | 3499.13 |
| gi|35298 | 2 | 86.06 | 0.02 | 1.65 | unnamed protein product [homo sapiens] | 2912.67 | 1766.95 |
| gi|35038 | 2 | 98.29 | 4.74E-03 | 1.64 | nuclear factor IV [homo sapiens] | 1902.46 | 1163.06 |
| gi|4503513 | 4 | 160.3 | 3.77E-06 | 1.63 | eukaryotic translation initiation factor 3 subunit I [homo sapiens] | 2.34E+04 | 3.81E+04 |
| gi|21614499 | 38 (2) | 1953.32 | 0.04 | 1.62 | ezrin [homo sapiens] | 2.86E+04 | 1.77E+04 |
| gi|230867 | 14 (2) | 1155.34 | 9.68E-03 | 1.62 | Chain R, Twinning In Crystals Of Human Skeletal Muscle D-Glyceraldehyde-3- Phosphate Dehydrogenase | 3.75E+05 | 2.31E+05 |
| gi|31397 | 5 (2) | 214.35 | 1.66E-06 | 1.62 | fibronectin precursor [homo sapiens] | 2.10E+04 | 1.29E+04 |
| gi|312137 | 7 (2) | 351.94 | 0.01 | 1.61 | fructose bisphosphate aldolase [homo sapiens] | 7275.15 | 1.17E+04 |
| gi|1531594 | 3 | 127.89 | 0.03 | 1.61 | unknown [homo sapiens] | 6392.49 | 1.03E+04 |
| gi|31831 | 3 (2) | 112.21 | 1.24E-03 | 1.61 | glutamate--ammonia ligase [homo sapiens] | 1.23E+04 | 7635.97 |
| gi|4826665 | 2 | 111.95 | 4.55E-03 | 1.6 | copper chaperone for superoxide dismutase [homo sapiens] | 1.40E+04 | 8720.91 |
| gi|132910 | 3 | 85.57 | 0.01 | 1.6 | RecName: Full=60S ribosomal protein L34 | 1.81E+04 | 1.13E+04 |
| gi|259090316 | 2 | 86.22 | 1.69E-03 | 1.59 | Chain A, Structure-Based Design Of Novel Pin1 Inhibitors (I) | 4523.03 | 7201.07 |
| gi|31542947 | 19 (8) | 1191.91 | 6.18E-05 | 1.58 | 60 kDa heat shock protein, mitochondrial [homo sapiens] | 4.26E+05 | 2.70E+05 |
| gi|5803013 | 7 (1) | 325.25 | 0.01 | 1.58 | endoplasmic reticulum resident protein 29 isoform 1 precursor [homo sapiens] | 8129.23 | 5137.1 |
| gi|8051631 | 5 | 271.6 | 6.24E-03 | 1.58 | RNA-binding protein Raly isoform 1 [homo sapiens] | 1.88E+04 | 1.19E+04 |
| gi|4508047 | 9 (8) | 474.55 | 1.02E-04 | 1.57 | zyxin [homo sapiens] | 8.57E+05 | 5.44E+05 |
| gi|5031877 | 16 (2) | 940.8 | 1.23E-03 | 1.56 | lamin-B1 isoform 1 [homo sapiens] | 1.21E+04 | 1.89E+04 |
| gi|125316 | 5 (2) | 150.12 | 0.03 | 1.55 | RecName: Full=Creatine kinase U-type, mitochondrial; AltName: Full=Acidic-type mitochondrial creatine kinase; Short=Mia-CK; AltName: Full=Ubiquitous mitochondrial creatine kinase; Short=U-MtCK; Flags: Precursor | 5524.55 | 3568.47 |
| gi|338634 | 2 | 157.55 | 8.04E-04 | 1.54 | syndecan [homo sapiens] | 1.16E+05 | 7.53E+04 |
| gi|7705927 | 3 | 151.38 | 1.34E-03 | 1.53 | ATPase inhibitor, mitochondrial isoform 1 precursor [homo sapiens] | 3.22E+04 | 2.11E+04 |
| gi|12231182 | 3 | 105.27 | 4.20E-04 | 1.53 | 65kDa FK506-binding protein [homo sapiens] | 1.34E+04 | 2.05E+04 |
| gi|18379349 | 2 | 102.89 | 6.09E-03 | 1.53 | synaptic vesicle membrane protein VAT-1 homolog [homo sapiens] | 1.08E+04 | 1.66E+04 |
| gi|4529893 | 27 (1) | 1684.36 | 0.03 | 1.52 | HSP70-1 [homo sapiens] | 3003.23 | 1981.42 |
| gi|987870 | 3 | 156.95 | 7.51E-03 | 1.52 | RNase L inhibitor [homo sapiens] | 9147.65 | 6019.4 |
| gi|3212355 | 3 (2) | 143.17 | 0.04 | 1.52 | Chain A, P11 (s100a10), Ligand Of Annexin Ii | 1.83E+04 | 2.78E+04 |
| gi|2895085 | 12 (3) | 643.76 | 5.43E-08 | 1.51 | hD54+ins2 isoform [homo sapiens] | 4.34E+04 | 2.87E+04 |
| gi|224586882 | 7 (2) | 432.23 | 6.86E-05 | 1.51 | Y-box-binding protein 3 isoform a [homo sapiens] | 3.20E+04 | 2.12E+04 |
| gi|87196339 | 11 (4) | 553.02 | 0.03 | 1.5 | collagen alpha-1(VI) chain precursor [homo sapiens] | 5.60E+04 | 8.41E+04 |
| gi|499719 | 7 | 417.29 | 3.12E-05 | 1.5 | mitochondrial dihydrolipoamide succinyltransferase [homo sapiens] | 1.62E+05 | 1.07E+05 |
| gi|116241354 | 4 (1) | 190.29 | 0.03 | 1.5 | RecName: Full=ES1 protein homolog, mitochondrial; AltName: Full=Protein GT335; AltName: Full=Protein KNP-I; Flags: Precursor | 4.46E+04 | 2.97E+04 |
| gi|20067392 | 3 | 94.6 | 2.46E-03 | 1.5 | thioredoxin related protein [homo sapiens] | 2.53E+04 | 1.69E+04 |
| gi|189306 | 24 (13) | 1301.56 | 3.27E-03 | 1.49 | nucleolin [homo sapiens] | 2.90E+05 | 1.95E+05 |
| gi|4826972 | 3 | 190.51 | 2.63E-04 | 1.49 | RNA-binding protein 8A [homo sapiens] | 4.24E+04 | 6.32E+04 |
| gi|182067 | 3 (1) | 162.95 | 0.03 | 1.49 | translational initiation factor beta subunit [homo sapiens] | 4253.85 | 2852.14 |
| gi|2996192 | 2 | 81.76 | 1.17E-03 | 1.49 | endobrevin [homo sapiens] | 6537.01 | 4382.99 |
| gi|1050527 | 2 (1) | 80.98 | 1.20E-03 | 1.49 | seryl-tRNA synthetase [homo sapiens] | 1444.72 | 970.02 |
| gi|913159 | 9 (5) | 544.42 | 1.84E-03 | 1.48 | neuropolypeptide h3 [human, brain, Peptide, 186 aa] | 6.15E+05 | 4.15E+05 |
| gi|11056044 | 9 (5) | 474.68 | 4.01E-03 | 1.48 | inorganic pyrophosphatase [homo sapiens] | 2.46E+05 | 1.66E+05 |
| gi|15928913 | 6 (3) | 373.02 | 0.01 | 1.48 | Unknown (protein for IMAGE:3906970), partial [homo sapiens] | 8.31E+04 | 5.61E+04 |
| gi|5453595 | 6 (5) | 339.89 | 8.46E-04 | 1.48 | adenylyl cyclase-associated protein 1 [homo sapiens] | 9.83E+04 | 6.62E+04 |
| gi|2392312 | 2 | 120.3 | 9.66E-03 | 1.48 | Chain A, Structure Of T255e, E376g Mutant Of Human Medium Chain Acyl- Coa Dehydrogenase | 9991.8 | 6761.83 |
| gi|2337920 | 3 | 209.2 | 2.53E-03 | 1.47 | syntaxin 7 [homo sapiens] | 5.27E+04 | 3.57E+04 |
| gi|8923001 | 3 | 207.84 | 2.79E-04 | 1.46 | mycophenolic acid acyl-glucuronide esterase, mitochondrial isoform 1 precursor [homo sapiens] | 4.93E+04 | 3.38E+04 |
| gi|4506663 | 4 | 186.12 | 5.24E-03 | 1.46 | 60S ribosomal protein L8 [homo sapiens] | 1.98E+04 | 1.36E+04 |
| gi|4557235 | 16 | 809.45 | 1.75E-03 | 1.45 | very long-chain specific acyl-CoA dehydrogenase, mitochondrial isoform 1 precursor [homo sapiens] | 1.62E+05 | 1.12E+05 |
| gi|5174539 | 3 | 171.62 | 0.03 | 1.45 | malate dehydrogenase, cytoplasmic isoform 2 [homo sapiens] | 1.35E+04 | 1.96E+04 |
| gi|3282771 | 7 (6) | 249.93 | 0.03 | 1.44 | actin-binding protein homolog ABP-278 [homo sapiens] | 4.20E+04 | 2.93E+04 |
| gi|14043072 | 24 (1) | 1543.74 | 2.21E-04 | 1.43 | heterogeneous nuclear ribonucleoproteins A2/B1 isoform B1 [homo sapiens] | 3.80E+04 | 5.41E+04 |
| gi|28277147 | 4 | 228.35 | 3.23E-05 | 1.43 | Metadherin [homo sapiens] | 5.57E+04 | 3.89E+04 |
| gi|5031753 | 2 | 142.8 | 2.57E-04 | 1.43 | heterogeneous nuclear ribonucleoprotein H [homo sapiens] | 1.11E+04 | 1.58E+04 |
| gi|10047203 | 2 (1) | 57.02 | 0.03 | 1.43 | KIAA1569 protein [homo sapiens] | 2.23E+04 | 1.56E+04 |
| gi|4504433 | 6 (1) | 361.69 | 0.04 | 1.42 | high mobility group protein HMG-I/HMG-Y isoform b [homo sapiens] | 4.59E+04 | 3.23E+04 |
| gi|18699732 | 6 | 290.19 | 4.08E-05 | 1.42 | 45 kDa calcium-binding protein isoform 2 precursor [homo sapiens] | 2.72E+04 | 1.91E+04 |
| gi|4200241 | 4 | 174.91 | 8.85E-03 | 1.42 | hypothetical protein [homo sapiens] | 3.93E+04 | 2.76E+04 |
| gi|4105190 | 2 (1) | 132.65 | 3.28E-03 | 1.41 | peroxisomal short-chain alcohol dehydrogenase [homo sapiens] | 2949.18 | 2094.61 |
| gi|181575 | 9 (2) | 521.33 | 0.02 | 1.4 | dihydrolipoamide dehydrogenase precursor [homo sapiens] | 1.64E+04 | 1.17E+04 |
| gi|6005890 | 5 | 262.09 | 7.41E-03 | 1.4 | transcription elongation factor B polypeptide 2 isoform a [homo sapiens] | 2.71E+04 | 3.80E+04 |
| gi|3916257 | 3 | 145.44 | 4.89E-03 | 1.4 | FK506-binding protein [homo sapiens] | 1.72E+04 | 2.42E+04 |
| gi|4507801 | 2 | 84.65 | 0.05 | 1.4 | small ubiquitin-related modifier 1 isoform a precursor [homo sapiens] | 2.39E+04 | 1.71E+04 |
| gi|4759212 | 7 (6) | 438.91 | 3.87E-04 | 1.38 | tubulin-specific chaperone A [homo sapiens] | 7.61E+04 | 1.05E+05 |
| gi|1224125 | 9 (8) | 338.21 | 0.02 | 1.38 | adenosine kinase [homo sapiens] | 3.92E+04 | 2.83E+04 |
| gi|46852390 | 7 (1) | 333.54 | 0.04 | 1.38 | coiled-coil domain-containing protein 6 [homo sapiens] | 2951.67 | 2143.88 |
| gi|895845 | 5 (4) | 237.44 | 7.19E-03 | 1.38 | p64 CLCP [homo sapiens] | 5.08E+04 | 7.04E+04 |
| gi|4502847 | 4 | 206.39 | 0.02 | 1.38 | cold-inducible RNA-binding protein [homo sapiens] | 3.89E+04 | 2.81E+04 |
| gi|4008131 | 13 (1) | 660.36 | 9.86E-03 | 1.37 | chaperonin 10 [homo sapiens] | 1.32E+05 | 9.62E+04 |
| gi|16924265 | 8 (5) | 542.81 | 6.37E-04 | 1.37 | Enoyl Coenzyme A hydratase 1, peroxisomal [homo sapiens] | 1.03E+05 | 7.53E+04 |
| gi|4503477 | 5 (3) | 249.7 | 8.37E-04 | 1.37 | elongation factor 1-beta [homo sapiens] | 6.09E+04 | 4.46E+04 |
| gi|13236495 | 5 | 170.14 | 6.27E-03 | 1.37 | quinone oxidoreductase isoform a [homo sapiens] | 9177.63 | 6677.54 |
| gi|6683817 | 4 | 144.42 | 1.28E-03 | 1.37 | MLL septin-like fusion protein MSF-A [homo sapiens] | 2.02E+04 | 1.47E+04 |
| gi|4503291 | 3 | 126.05 | 2.14E-03 | 1.37 | D-dopachrome decarboxylase [homo sapiens] | 6.02E+04 | 4.39E+04 |
| gi|16877641 | 4 | 109.7 | 1.75E-03 | 1.37 | Proline-rich coiled-coil 1 [homo sapiens] | 1.11E+04 | 8071.78 |
| gi|22208967 | 7 (2) | 404.72 | 0.02 | 1.36 | high mobility group protein HMG-I/HMG-Y isoform a [homo sapiens] | 1.89E+05 | 1.39E+05 |
| gi|179531 | 8 (4) | 377.81 | 0.04 | 1.36 | IgE-binding protein [homo sapiens] | 3.06E+04 | 2.24E+04 |
| gi|558349 | 3 (2) | 75.73 | 7.37E-03 | 1.36 | host cell factor [homo sapiens] | 7792.86 | 5732.1 |
| gi|4505701 | 6 | 324.16 | 3.84E-05 | 1.35 | pyridoxal kinase [homo sapiens] | 6.02E+04 | 4.45E+04 |
| gi|4506707 | 4 (1) | 197.55 | 0.02 | 1.35 | 40S ribosomal protein S25 [homo sapiens] | 2.27E+04 | 1.68E+04 |
| gi|3098601 | 3 (2) | 192.46 | 0.02 | 1.35 | Ras-GAP SH3 binding protein [homo sapiens] | 1.61E+04 | 1.19E+04 |
| gi|56554357 | 11 (7) | 613.83 | 6.88E-04 | 1.34 | Chain A, Binary Structure Of Human Decr Solved By Semet Sad. | 3.51E+05 | 2.62E+05 |
| gi|190804 | 3 (1) | 174.82 | 0.01 | 1.34 | ubiquinone-binding protein [homo sapiens] | 1.17E+04 | 8725.7 |
| gi|9955330 | 11 (2) | 542.62 | 0.01 | 1.33 | Chain A, Sterol Carrier Protein-2, Nmr, 20 Structures | 5.61E+04 | 4.23E+04 |
| gi|4506195 | 6 (5) | 406.92 | 6.20E-03 | 1.33 | proteasome subunit beta type-2 isoform 1 [homo sapiens] | 7.22E+04 | 5.43E+04 |
| gi|348239 | 5 (4) | 205.53 | 8.98E-03 | 1.33 | unnamed protein product [homo sapiens] | 1.67E+04 | 1.26E+04 |
| gi|14714625 | 2 | 96.68 | 5.35E-05 | 1.33 | Papillary renal cell carcinoma (translocation-associated) [homo sapiens] | 1.11E+04 | 8330.53 |
| gi|10947135 | 2 | 79.98 | 4.39E-03 | 1.33 | ATP-binding cassette sub-family F member 1 isoform b [homo sapiens] | 7535.75 | 5675.22 |
| gi|14211923 | 2 | 97.03 | 0.04 | 1.32 | histidine triad nucleotide-binding protein 2, mitochondrial precursor [homo sapiens] | 1.56E+04 | 2.06E+04 |
| gi|440308 | 11 (9) | 612.93 | 2.93E-05 | 1.31 | enhancer protein [homo sapiens] | 3.46E+05 | 2.64E+05 |
| gi|10835067 | 9 (6) | 431.3 | 7.38E-03 | 1.31 | lupus La protein [homo sapiens] | 2.82E+04 | 2.16E+04 |
| gi|13569956 | 2 | 119.35 | 0.02 | 1.31 | actin-related protein 2/3 complex subunit 5-like protein [homo sapiens] | 1.75E+04 | 1.34E+04 |
| gi|284164 | 2 | 99.79 | 4.73E-03 | 1.31 | arginine-rich protein - human | 1.63E+04 | 1.24E+04 |
| gi|118498356 | 25 (15) | 1370.26 | 0.02 | 1.3 | kinectin isoform a [homo sapiens] | 1.11E+05 | 1.44E+05 |
| gi|182087 | 15 (13) | 716.52 | 5.30E-03 | 1.3 | amplaxin [homo sapiens] | 1.10E+05 | 8.47E+04 |
| gi|4503143 | 10 (7) | 499.29 | 0.04 | 1.3 | cathepsin D preproprotein [homo sapiens] | 1.65E+05 | 1.27E+05 |
| gi|704348 | 3 | 260.15 | 0.02 | 1.3 | paxillin [homo sapiens] | 6.38E+04 | 4.91E+04 |
| gi|4506387 | 8 (7) | 335.72 | 9.21E-03 | 1.29 | UV excision repair protein RAD23 homolog B isoform 1 [homo sapiens] | 1.63E+05 | 1.27E+05 |
| gi|5901926 | 7 (6) | 291.91 | 0.03 | 1.29 | cleavage and polyadenylation specificity factor subunit 5 [homo sapiens] | 3.60E+04 | 2.78E+04 |
| gi|21730367 | 4 | 223.74 | 0.02 | 1.29 | Chain A, Ca2+-Binding Mimicry In The Crystal Structure Of The Eu3+-Bound Mutant Human Macrophage Capping Protein Cap G | 2.76E+04 | 2.14E+04 |
| gi|6331231 | 3 | 108.88 | 0.01 | 1.29 | KIAA1271 protein [homo sapiens] | 4.47E+04 | 3.46E+04 |
| gi|14042346 | 2 | 81.07 | 0.04 | 1.29 | unnamed protein product [homo sapiens] | 4035.21 | 3136.77 |
| gi|3264861 | 4 | 153.84 | 0.01 | 1.28 | eukaryotic translation initiation factor eIF3, p35 subunit [homo sapiens] | 1.98E+04 | 1.54E+04 |
| gi|163644321 | 2 | 57.75 | 0.02 | 1.28 | cytochrome b-c1 complex subunit Rieske, mitochondrial [homo sapiens] | 8201.72 | 6397.11 |
| gi|443382 | 11 (6) | 558.6 | 0.01 | 1.27 | Chain A, Structure Of Inositol Monophosphatase, The Putative Target Of Lithium Therapy | 6.55E+04 | 5.18E+04 |
| gi|1942335 | 6 | 422.21 | 0.02 | 1.27 | Chain C, Ternary Complex Of A Calcineurin A Fragment, Calcineurin B, Fkbp12 And The Immunosuppressant Drug Fk506 (tacrolimus) | 5.61E+05 | 4.42E+05 |
| gi|5031593 | 4 | 210.62 | 0.03 | 1.27 | actin-related protein 2/3 complex subunit 5 isoform 1 [homo sapiens] | 6.41E+04 | 5.05E+04 |
| gi|16550968 | 3 | 152.92 | 0.02 | 1.27 | unnamed protein product [homo sapiens] | 1.35E+04 | 1.06E+04 |
| gi|32129199 | 5 | 298.37 | 0.01 | 1.26 | SAP domain-containing ribonucleoprotein [homo sapiens] | 8.05E+04 | 6.38E+04 |
| gi|8923812 | 3 | 156.85 | 0.02 | 1.26 | acyl-coenzyme A thioesterase 13 isoform 1 [homo sapiens] | 1.57E+04 | 1.24E+04 |
| gi|2160784 | 44 (41) | 2672.92 | 0.03 | 1.25 | myasthenia gravis autoantigen gravin [homo sapiens] | 4.98E+05 | 6.20E+05 |
| gi|338221409 | 39 (25) | 2135.41 | 4.27E-05 | 1.25 | p180/ribosome receptor [homo sapiens] | 1.55E+05 | 1.24E+05 |
| gi|4502297 | 4 (2) | 235.91 | 0.04 | 1.25 | ATP synthase subunit delta, mitochondrial precursor [homo sapiens] | 6.78E+04 | 5.41E+04 |
| gi|41327741 | 4 | 228.92 | 0.02 | 1.25 | persulfide dioxygenase ETHE1, mitochondrial [homo sapiens] | 3.67E+04 | 2.93E+04 |
| gi|190786 | 4 | 133.6 | 0.04 | 1.25 | prolyl 4-hydroxylase alpha subunit (EC 1.14.11.2) [homo sapiens] | 2.03E+04 | 2.53E+04 |
| gi|21361670 | 6 (4) | 334.18 | 1.08E-03 | 1.24 | drebrin-like protein isoform a [homo sapiens] | 3.71E+04 | 4.59E+04 |
| gi|4506339 | 2 | 142.43 | 6.33E-03 | 1.24 | peroxisomal biogenesis factor 19 isoform a [homo sapiens] | 1.61E+04 | 1.30E+04 |
| gi|194387612 | 3 (2) | 102.28 | 3.87E-03 | 1.24 | unnamed protein product [homo sapiens] | 1.27E+04 | 1.03E+04 |
| gi|4507877 | 34 (30) | 1712.96 | 0.02 | 1.23 | vinculin isoform VCL [homo sapiens] | 3.28E+05 | 4.04E+05 |
| gi|4557553 | 2 | 120.06 | 0.04 | 1.23 | emerin [homo sapiens] | 9006.34 | 1.11E+04 |
| gi|20162550 | 2 | 109.83 | 7.66E-03 | 1.23 | protein phosphatase 1 regulatory subunit 14B [homo sapiens] | 7.57E+04 | 6.14E+04 |
| gi|5031875 | 36 (33) | 2119.48 | 0.03 | 1.22 | lamin isoform C [homo sapiens] | 4.52E+05 | 5.52E+05 |
| gi|5803225 | 23 (20) | 1304.6 | 7.25E-03 | 1.22 | 14-3-3 protein epsilon [homo sapiens] | 3.64E+05 | 4.45E+05 |
| gi|9966827 | 7 (4) | 430.42 | 0.03 | 1.22 | PEST proteolytic signal-containing nuclear protein [homo sapiens] | 4.88E+04 | 4.00E+04 |
| gi|4506455 | 6 (1) | 387.22 | 0.01 | 1.22 | reticulocalbin-1 precursor [homo sapiens] | 2.73E+04 | 2.23E+04 |
| gi|189308 | 7 (6) | 378.38 | 4.82E-04 | 1.21 | nucleobindin [homo sapiens] | 5.25E+04 | 4.34E+04 |
| gi|194306653 | 6 | 368.19 | 0.02 | 1.21 | yorkie homolog isoform 1 [homo sapiens] | 7.83E+04 | 6.46E+04 |
| gi|5453832 | 7 | 351.38 | 0.04 | 1.21 | hypoxia up-regulated protein 1 precursor [homo sapiens] | 3.23E+04 | 2.68E+04 |
| gi|5174613 | 6 (3) | 364.33 | 7.07E-03 | 1.2 | nucleosome assembly protein 1-like 4 [homo sapiens] | 3.68E+04 | 4.43E+04 |
| gi|4506243 | 8 | 349.75 | 0.05 | 1.2 | polypyrimidine tract-binding protein 1 isoform a [homo sapiens] | 8.66E+04 | 7.24E+04 |
| gi|4759140 | 4 | 174.42 | 0.04 | 1.2 | Na(+)/H(+) exchange regulatory cofactor NHE-RF1 [homo sapiens] | 4.23E+04 | 3.54E+04 |
| gi|4507793 | 5 | 288.44 | 4.08E-03 | 1.19 | ubiquitin-conjugating enzyme E2 N [homo sapiens] | 7.65E+04 | 9.11E+04 |
| gi|29653 | 2 | 57.42 | 0.05 | 1.19 | putative oncogene [homo sapiens] | 4272.03 | 3581.84 |
| gi|840771 | 2 | 153.74 | 0.04 | 1.18 | leucocyte antigen CD97 [homo sapiens] | 2.02E+04 | 1.71E+04 |
| gi|394986254 | 5 | 261.15 | 0.03 | 1.17 | Chain A, X-Ray Structure Of Human Soul | 8.22E+04 | 7.03E+04 |
| gi|4503727 | 11 (10) | 658.12 | 0.04 | 1.16 | peptidyl-prolyl cis-trans isomerase FKBP3 [homo sapiens] | 1.47E+05 | 1.70E+05 |
| gi|109637759 | 11 | 472.65 | 8.77E-03 | 1.16 | calpastatin isoform f [homo sapiens] | 6.77E+04 | 5.82E+04 |
| gi|1584035 | 10 (9) | 464.05 | 0.05 | 1.16 | Lasp-1 protein | 2.16E+05 | 1.86E+05 |
