# Supplementary material for: Proteomic identification of the lactate dehydrogenase A in a radioresistant prostate cancer xenograft mouse model for improving radiotherapy
Source: Oncotarget. 2016 Sep 30;7(45):74269–85. doi: 10.18632/oncotarget.12368 (PMC5342052; doi:10.18632/oncotarget.12368)
Supplement: Supplementary file 3 [file oncotarget-07-74269-s003.doc]

**Table S3. Significant signaling pathways associated with CaP radioresistance identified by Ingenuity Pathways Analysis**

| **Pathways** | **CaP**  **Publications** | **-log(p-value)** | **Ratio** | **Molecules** | **Molecules number** |
| --- | --- | --- | --- | --- | --- |
| VEGF Signaling | 302 | 2.95 | 6.52E-02 | SFN,VCL,ACTG1,EIF2S2,PXN,YWHAE | 6 |
| Integrin Signaling | 260 | 4.99 | 5.97E-02 | ARPC2,ZYX,ARPC5,VCL,ACTG1,CTTN,RAP1B,PXN,ARPC5L,ACTR3,RAC1,ITGB4 | 12 |
| IGF-1 Signaling | 212 | 2.84 | 6.19E-02 | SFN,YWHAG,PXN,YWHAE,YWHAZ,YWHAH | 6 |
| Glycolysis I | 140 | 4.87 | 2E-01 | PGK1,ALDOC,PKM,GAPDH,ALDOA | 5 |
| Protein Kinase A Signaling | 96 | 2.42 | 3.12E-02 | FLNB,GNAI2,AKAP12,SFN,YWHAG,PPP1R14B,RAP1B,PXN,PPP3CA,YWHAE,YWHAZ,YWHAH | 12 |
| Mitochondrial Dysfunction | 90 | 2.81 | 4.68E-02 | CAT,NDUFV3,ATP5J,ATP5A1,UQCRFS1,ATP5D,ATP5B,UQCRB | 8 |
| Coagulation System | 72 | 2.01 | 8.57E-02 | PLAU,SERPINA1,SERPINC1 | 3 |
| Oxidative Phosphorylation | 71 | 3.32 | 6.42E-02 | NDUFV3,ATP5J,ATP5A1,UQCRFS1,ATP5D,ATP5B,UQCRB | 7 |
| Actin Cytoskeleton Signaling | 61 | 4.66 | 5.53E-02 | ARPC2,ARPC5,MYH1,FN1,VCL,ACTG1,GSN,EZR,PXN,ARPC5L,ACTR3,RAC1 | 12 |
| RhoA Signaling | 59 | 3.79 | 6.56E-02 | ARPC2,ARPC5,ACTG1,KTN1,EZR,SEPT9,ARPC5L,ACTR3 | 8 |
| Protein Ubiquitination Pathway | 56 | 4.63 | 5.1E-02 | PSMB6,TCEB2,UBA1,HSPA1A/HSPA1B,HSPD1,UBE2N,B2M,HSPE1,HSPB1,HSPA4,HLA-A,PSMB2,PSMB3 | 13 |
| p70S6K Signaling | 48 | 3.1 | 5.88E-02 | GNAI2,SFN,YWHAG,YWHAE,YWHAZ,YWHAH,EEF2 | 7 |
| Unfolded protein response | 43 | 2.32 | 7.41E-02 | HSPA4,HSPA1A/HSPA1B,CANX,VCP | 4 |
| TCA Cycle II (Eukaryotic) | 33 | 3.74 | 1.74E-01 | DLD,FH,DLST,MDH1 | 4 |
| Paxillin Signaling | 26 | 2.04 | 4.95E-02 | VCL,ACTG1,PXN,RAC1,ITGB4 | 5 |
| Glycogen Degradation III | 26 | 2.01 | 1.67E-01 | PGM1,GAA | 2 |
| Ephrin Receptor Signaling | 21 | 2.77 | 4.6E-02 | ARPC2,ARPC5,GNAI2,RAP1B,PXN,ARPC5L,ACTR3,RAC1 | 8 |
| ILK Signaling | 18 | 2.59 | 4.3E-02 | FLNB,MYH1,FN1,ACTG1,PPP1R14B,PXN,ITGB4,KRT18 | 8 |
| Myc Mediated Apoptosis Signaling | 17 | 3.08 | 8.62E-02 | SFN,YWHAG,YWHAE,YWHAZ,YWHAH | 5 |
| Signaling by Rho Family GTPases | 15 | 2.52 | 3.85E-02 | ARPC2,ARPC5,GNAI2,ACTG1,EZR,SEPT9,ARPC5L,ACTR3,RAC1 | 9 |
| Creatine-phosphate Biosynthesis | 14 | 3.03 | 5E-01 | CKMT2,CKMT1A/CKMT1B | 2 |
| Gluconeogenesis I | 13 | 4.87 | 2E-01 | PGK1,ALDOC,GAPDH,ALDOA,MDH1 | 5 |
| Antigen Presentation Pathway | 13 | 2.92 | 1.08E-01 | PSMB6,HLA-A,CANX,B2M | 4 |
| Atherosclerosis Signaling | 12 | 2.3 | 4.84E-02 | APOA4,CLU,APOA2,APOA1,SERPINA1,ALB | 6 |
| Epithelial Adherens Junction Signaling | 10 | 4.76 | 6.85E-02 | ARPC2,ZYX,ARPC5,MYH1,VCL,ACTG1,RAP1B,ARPC5L,ACTR3,RAC1 | 10 |
| ERK/MAPK Signaling | 9 | 2.57 | 4.28E-02 | HSPB1,YWHAG,PPP1R14B,RAP1B,PXN,YWHAZ,YWHAH,RAC1 | 8 |
| HIPPO signaling | 8 | 3.96 | 8.14E-02 | SFN,YWHAG,PPP1R14B,YAP1,YWHAE,YWHAZ,YWHAH | 7 |
| ERK5 Signaling | 8 | 2.92 | 7.94E-02 | SFN,YWHAG,YWHAE,YWHAZ,YWHAH | 5 |
| Acute Phase Response Signaling | 8 | 2.85 | 4.73E-02 | ITIH4,FN1,APCS,APOA2,APOA1,SERPINA1,AHSG,ALB | 8 |
| Cell Cycle: G2/M DNA Damage Checkpoint Regulation | 5 | 3.42 | 1.02E-01 | SFN,YWHAG,YWHAE,YWHAZ,YWHAH | 5 |
| EIF2 Signaling | 5 | 2.6 | 4.32E-02 | RPL8,EIF2S2,RPL7A,RPL14,EIF3I,EIF3J,RPL19,RPS25 | 8 |
| Clathrin-mediated Endocytosis Signaling | 4 | 7.84 | 8.11E-02 | APOA4,CTTN,ARPC5L,PPP3CA,ACTR3,ARPC2,ARPC5,ACTG1,CLU,APOA2,APOA1,SERPINA1,RAC1,ITGB4,ALB | 15 |
| Amyotrophic Lateral Sclerosis Signaling | 4 | 2.81 | 6.12E-02 | GLUL,CAT,GPX1,PPP3CA,RAC1,CCS | 6 |
| RhoGDI Signaling | 2 | 3.43 | 5.2E-02 | GDI1,ARPC2,ARPC5,GNAI2,ACTG1,EZR,ARPC5L,ACTR3,RAC1 | 9 |
| Remodeling of Epithelial Adherens Junctions | 1 | 5.65 | 1.18E-01 | ARPC2,ZYX,ARPC5,VCL,ACTG1,MAPRE1,ARPC5L,ACTR3 | 8 |
| Regulation of Actin-based Motility by Rho | 1 | 2.98 | 6.59E-02 | ARPC2,ARPC5,GSN,ARPC5L,ACTR3,RAC1 | 6 |
| IL-12 Signaling and Production in Macrophages | 1 | 2.13 | 4.44E-02 | APOA4,CLU,APOA2,APOA1,SERPINA1,ALB | 6 |
| Fcγ Receptor-mediated Phagocytosis in Macrophages and Monocytes | 0 | 4.63 | 8.6E-02 | ARPC2,ARPC5,ACTG1,EZR,PXN,ARPC5L,ACTR3,RAC1 | 8 |
| FXR/RXR Activation | 0 | 4.46 | 7.09E-02 | ITIH4,APOA4,CLU,APOA2,APOA1,SERPINA1,AHSG,ALB,SDC1 | 9 |
| Production of Nitric Oxide and Reactive Oxygen Species in Macrophages | 0 | 3.99 | 5.56E-02 | APOA4,PPP1R14B,CLU,CAT,APOA2,RAP1B,APOA1,SERPINA1,RAC1,ALB | 10 |
| LXR/RXR Activation | 0 | 3.81 | 6.61E-02 | ITIH4,APOA4,CLU,APOA2,APOA1,SERPINA1,AHSG,ALB | 8 |
| Caveolar-mediated Endocytosis Signaling | 0 | 3.56 | 8.45E-02 | FLNB,HLA-A,ACTG1,B2M,ITGB4,ALB | 6 |
| fMLP Signaling in Neutrophils | 0 | 3.35 | 6.48E-02 | ARPC2,ARPC5,GNAI2,ARPC5L,PPP3CA,ACTR3,RAC1 | 7 |
| Actin Nucleation by ARP-WASP Complex | 0 | 3.15 | 8.93E-02 | ARPC2,ARPC5,ARPC5L,ACTR3,RAC1 | 5 |
| Virus Entry via Endocytic Pathways | 0 | 3.03 | 6.74E-02 | FLNB,HLA-A,ACTG1,B2M,RAC1,ITGB4 | 6 |
| 2-ketoglutarate Dehydrogenase Complex | 0 | 3.03 | 5E-01 | DLD,DLST | 2 |
| 14-3-3-mediated Signaling | 0 | 2.43 | 5.13E-02 | SFN,YWHAG,YAP1,YWHAE,YWHAZ,YWHAH | 6 |
| Leukocyte Extravasation Signaling | 0 | 2.42 | 4.04E-02 | GNAI2,VCL,ACTG1,CTTN,EZR,RAP1B,PXN,RAC1 | 8 |
| CD28 Signaling in T Helper Cells | 0 | 2.41 | 5.08E-02 | ARPC2,ARPC5,ARPC5L,PPP3CA,ACTR3,RAC1 | 6 |
| Sucrose Degradation V (Mammalian) | 0 | 2.27 | 2.22E-01 | ALDOC,ALDOA | 2 |
| Regulation of Cellular Mechanics by Calpain Protease | 0 | 2.23 | 7.02E-02 | VCL,EZR,PXN,CAST | 4 |
